# Going beyond SMILES enumeration for data augmentation in generative drug discovery

**DOI:** 10.1039/d5dd00028a

**Published:** 2025-08-14

**Authors:** Helena Brinkmann, Antoine Argante, Hugo ter Steege, Francesca Grisoni

**Affiliations:** a Institute for Complex Molecular Systems (ICMS), Eindhoven AI Systems Institute (EAISI), Department of Biomedical Engineering, Eindhoven University of Technology Eindhoven The Netherlands f.grisoni@tue.nl; b Centre for Living Technologies, Alliance TU/e, WUR, UU, UMC Utrecht Utrecht The Netherlands

## Abstract

Data augmentation can alleviate the limitations of small molecular datasets for generative deep learning by ‘artificially inflating’ the number of instances available for training. SMILES enumeration – wherein multiple valid SMILES strings are used to represent the same molecules – has become particularly beneficial to improve the quality of *de novo* molecule design. Herein, we investigated whether rethinking SMILES augmentation techniques could further enhance the quality of *de novo* design. To this end, we introduce four novel approaches for SMILES augmentation, drawing inspiration from natural language processing and chemistry insights: (a) token deletion, (b) atom masking, (c) bioisosteric substitution, and (d) self-training. *Via* systematic analysis, our results showed the promise of considering additional strategies for SMILES augmentation. Every strategy showed distinct advantages; for example, atom masking is particularly promising to learn desirable physico-chemical properties in very low-data regimes, and deletion to create novel scaffolds. This new repertoire of SMILES augmentation strategies expands the available toolkit to design molecules with bespoke properties in low-data scenarios.

## Abstract 2

We propose novel SMILES augmentation strategies for chemical language modelling, which broaden the range of tools for generative drug design.

## Graphical abstract

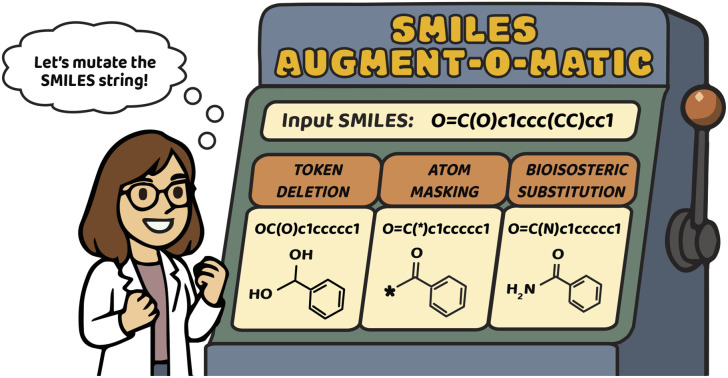

## Introduction

The chemical universe of drug-like molecules is incredibly vast, making the discovery of new medicinal drugs with traditional approaches a daunting task.^1^ Generative deep learning has gained remarkable attention due to its ability to generate molecules on-demand with desirable properties. Notably, chemical language models^2^ (CLMs) have shown their potential to learn complex molecular properties^3–5^ and have been applied to numerous wet-lab studies for bioactive ligand design.^5–8^ CLMs adapt algorithms from natural language processing (NLP) to learn the ‘chemical language’ and generate molecules in the form of strings with desirable properties.^2^

**Fig. 1:**
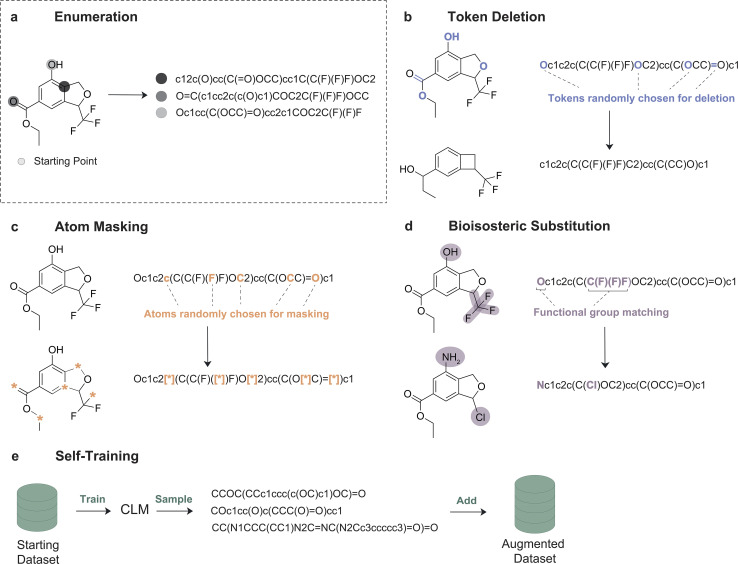
Overview of SMILES augmentation methods. (a) SMILES enumeration^28^ (used as a baseline in this work), where multiple SMILES strings are obtained by starting the graph traversal from different non-hydrogen atoms and/or by proceeding in different directions. (b) Token deletion, where new SMILES strings are generated by randomly removing tokens from the original string. (c) Atom masking, where atoms are randomly replaced with dummy tokens (‘[*]’). (d) Bioisosteric substitution, where pre-defined functional groups are substituted with their reported bioisosteres. (e) Self-training, where novel SMILES are generated by a trained CLM and used in turn to the initial set for the next training phase.

Simplified molecular input line entry system (SMILES)^9^ strings are one of the most widely used line notations for CLMs.^2,10–13^ SMILES strings represent two-dimensional molecular information in the form of text (Fig. 1a) by traversing the molecular graph and annotating (topo)chemical information with dedicated characters (‘tokens’) that represent atoms, bonds, rings, and branches. SMILES are non-univocal: the same molecule can be represented with different SMILES strings, depending on the starting atom and the chosen graph traversal path (Fig. 1a). Such non-univocity becomes beneficial to achieve data augmentation,^14^*i.e.*, to artificially inflate the number of samples available for training ‘data-hungry’ CLMs. *Via* SMILES enumeration (also referred to as ‘randomization’^14^), a molecule is represented by several different SMILES strings during training. SMILES enumeration yields beneficial effects on the quality of *de novo* drug designs,^15,16^ especially in low-data scenarios.^17,18^ Moreover, SMILES enumeration has improved model quality in various other chemistry tasks, *e.g*., organic synthesis planning,^19,20^ bioactivity prediction,^21,22^ and supramolecular chemistry.^23^

Inspired by the impact of SMILES enumeration, we introduce additional augmentation strategies to further stretch the boundaries of chemical language modelling. In this work, we adopted a broad definition of data augmentation from the NLP domain – namely, as a set of strategies for increasing the diversity and number of training examples without explicitly collecting new data.^24^ This can be achieved by “adding slightly modified copies of existing data or generating synthetic data from existing data”.^25^ By combining augmentation techniques inspired by NLP^25^ with chemistry insights, herein, we introduce, for the first time, four SMILES augmentation strategies for *de novo* design, extending from identity-preserving to identity-altering augmentations: (a) *token deletion*, whereby specific tokens are removed from a SMILES string; (b) *atom masking*, which replaces specific atoms with a placeholder token; (c) *bioisosteric substitution*, which replaces functional groups with their corresponding bioisosteres,^26^ and (d) *self-training*, where SMILES strings generated by a CLM are used as input for the next training phase. These approaches, in several variants, were systematically compared to SMILES enumeration, with varying training set sizes and in combination with transfer learning.

Our results show the distinct advantages of each augmentation strategy, for example, the potential of atom masking as a good alternative to SMILES enumeration, in particular low-data scenarios, for distribution learning or deletion for design of structurally diverse candidates. Ultimately, our work equips machine learning practitioners with a broader computational toolkit for chemical space exploration with CLMs.

## Results and discussion

### Novel data augmentation approaches

In this work, we investigated four strategies for SMILES augmentation (Fig. 1):

• **Token Deletion** (Fig. 1b), which removes specific symbols (‘tokens’) from a SMILES string to generate variations in the original input. We performed three deletion strategies:

• *Random deletion*, whereby tokens are randomly removed from a given string. A similar approach has been explored for molecular property prediction.^27^

• *Random deletion with enforced validity*, whereby, after randomly removing tokens, only ‘chemically valid’ SMILES strings are retained.

• *Random deletion with protection*, whereby only certain types of tokens are subjected to deletion. In particular, we protected ring- and branching-related tokens, whose incorrect notation is a failure mode of CLMs.^4^

The deletion of tokens for each variant was controlled by a probability of deletion (*p*).

• **Atom Masking** (Fig. 1c), which replaces specific atoms with a placeholder (‘mask’). We investigated two token masking strategies:

• *Random masking*, whereby randomly selected atoms are replaced by a dummy token (‘*’, Fig. 1c). A similar strategy was explored for molecular property prediction.^27^

• *Masking of functional groups*, whereby atoms belonging to pre-defined functional groups are masked. This is based on the hypothesis that masking functional groups might improve the learning of the ‘chemical semantics’ compared to random masking. A pre-defined list of ‘chemically relevant’ functional groups was used (Supporting Fig. S1).^29^

In both cases, the probability of atoms getting masked is controlled by a user-defined probability (*p*). Unlike commonly used masking approaches (*e.g*., in transformer-based methods^30,31^), the aim here is not to predict the masked input, but to introduce noise into the data to potentially increase robustness and generalizability.

• **Bioisosteric substitution** (Fig. 1d), which replaces groups of tokens with their respective bioisosteres. Bioisosteres – chemical groups that can be interchanged in a molecule while preserving its biological properties – are a key concept in medicinal chemistry.^26^ In this work, pre-defined functional groups (same as in atom masking) were replaced with the corresponding bioisosteres (if any), as reported in the SwissBioisostere Database.^32^ Functional groups were replaced by choosing randomly among their subset of top-5 frequently reported bioisosteres (see Materials and methods). The replacement was controlled by a user-defined probability (*p*).

• **Augmentation by self-training** (Fig. 1e). We define self-training as the process of feeding a generative deep learning approach its own generated samples. Here, we created ‘synthetic’ SMILES strings by sampling from a trained CLM on non-augmented SMILES strings, to be used to augment the training set available (for the follow-up training). This was achieved by temperature sampling of a trained CLM using a low temperature value (*T* = 0.5, see Materials and ethods, eqn (1)).

For each strategy, the augmented SMILES strings were used as input of the CLM for training. For chemical language modelling, we used a recurrent neural network with long short-term memory,^33,34^ which has found widespread applications in drug design and in combination with SMILES enumeration.^6,10,11,18,34^

### Method performance across dataset sizes

We analysed the performance of each method across data size scenarios, focusing on the ability to learn the ‘chemical syntax’ of the SMILES language and the physico-chemical properties of the training set. For each augmentation strategy, we trained CLMs using (a) three levels of probability of perturbation (*p* = 0.05, *p* = 0.15 and *p* = 0.30) for token deletion, atom masking, and bioisosteric substitution; (b) four levels of augmentation, *i.e.*, one-fold (no augmentation), three-, five- and ten-fold augmentation (corresponding to using three, five, and ten times more SMILES than the original training set size, respectively); and (c) five training sets extracted from ChEMBL,^35^ and containing different numbers of molecules (1000, 2500, 5000, 7500, and 10 000 molecules). Not all methods could augment until the wanted fold, and therefore were augmented until their possible maximum (Supporting Table S1). Enumeration was used as a baseline to benchmark the potential of the new augmentation strategies; for this method, ten-fold augmentation was used based on its performance (Supporting Fig. S2). For each setup, a CLM was trained on the (augmented) set and used to generate 1000 SMILES across three repeats (3000 generated strings in total) in a next-token prediction approach.

First, we evaluated the ability to learn the ‘chemical syntax’ of the SMILES language. We evaluated the generated SMILES strings based on: (a) validity, the percentage of SMILES strings that can be mapped back to ‘chemically valid’ molecules; (b) uniqueness, the percentage of non-duplicated molecules within the sampled set; and (c) novelty, the percentage of *de novo* designs that are not included in the training sets. For conciseness, here we report the results of 3-fold and 10-fold augmentation, while the remaining results can be found in SI Fig. S2.

Varying the perturbation probability *p* had a moderate but non-negligible effect on the validity of the generated strings (Supporting Fig. S2) and little to no effect on the uniqueness and novelty values (Supporting Fig. S3 and 4). Each method showed optimal probability values to maximize validity (token deletion and random masking: *p* = 0.05, bioisosteric substitution: *p* = 0.15; functional group masking: *p* = 0.30; Supporting Fig. S2), which will be used for the remainder of this work.

**Fig. 2:**
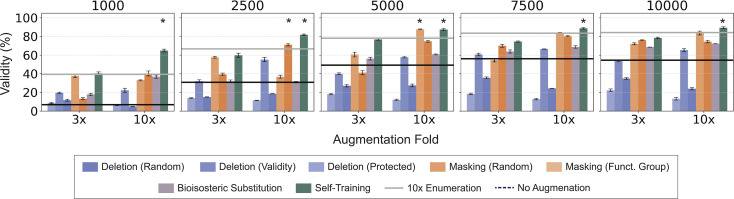
Syntactic validity of SMILES across augmentation strategies and augmentation folds. Several folds of augmentation (three- and ten-folds), across five training set sizes (1000, 2500, 5000, 75 000, and 10 000 SMILES) were analyzed. For each set-up, 1000 SMILES strings were generated across four repetitions for the analysis. The highest validity obtained by SMILES enumeration and without any augmentation is represented as solid and dashed lines, respectively. Statistically significant differences (one-sided Wilcoxon rank-sum test, *p* < 0.05) between the new augmentation approaches and SMILES enumeration (10×) are marked with asterisks.

All methods, except for random and protected token deletion, achieve a higher validity compared to the baseline without augmentation (Fig. 2). The beneficial effect of the augmentation strategies depends on (a) the augmentation fold – the higher, the better in general, and (b) the training set size – the higher, the lower the effect on validity, as previously reported for SMILES enumeration.^10^ The validity achieved by token deletion declines or plateaus with increasing dataset size, owing to the effect of model training with invalid and/or less common SMILES strings (as particularly visible with high *p* values and augmentation folds). Only self-training augmentation performs better than enumeration for all dataset sizes for 10× augmentation (one-sided Wilcoxon rank-sum test, *p* < 0.05).

On uniqueness and novelty, fewer differences among methods exist (Supporting Fig. S3 and 4), and almost all methods achieve values close to 100%. Atom masking yielded lower uniqueness and novelty values than the other approaches (up to 78.9% worse, SI Fig. S3), possibly owing to the artificial token ‘*‘, which might bias the model towards learning and reproducing patterns already seen in the training data. There is no clear evidence if higher probability works better or worse for atom masking in general.

Next, we evaluated each augmentation method for its ability to match the physico-chemical properties of the training set (‘distribution learning’). To this end, we computed eight properties: number of aliphatic and aromatic rings, molecular weight (MW), octanol–water partition coefficient (log *P*), number of hydrogen bond donors (HBD) and acceptors (HBA), topological polar surface area (TPSA), and number of rotatable bonds. The similarity between the training set and the *de novo* designs was measured *via* the Kolmogorov–Smirnov (KS) distance^36^ (the lower, the higher the similarity).

The results depend on the property being analysed (Table 1: HBA, HBD, MW, and log *P*; Supporting Table S1: number of aliphatic and aromatic rings and of rotatable bonds, and TPSA). Moreover, the distribution learning ability depends on the size of the training set (Table 1 and Table S1). Smaller training sets (1000 and 2500 molecules) yielded mostly higher KS values than the bigger ones (5000 molecules and above), highlighting the difficulty in learning property distribution properties from limited data. Certain properties (*i.e.*, number of aliphatic rings, and hydrogen bond donor) were less affected by the augmentation strategy, with no clear property-augmentation trends.

**Table 1:** Distribution learning of physico-chemical properties. We report the Kolmogorov–Smirnov (KS) distance between the *de novo* designs (3000 SMILES strings) and the training set molecules, computed for selected descriptors (HBA = number of hydrogen bond acceptors, HBD = number of hydrogen bond donors, MW = molecular weight, and log *P* = octanol–water partitioning coefficient). For each training set size (1000, 2500, 5000, 7500, and 10 000 molecules), the KS distance is reported for each augmentation strategy and each descriptor. For each descriptor and training set size, the best and second-best KS distances are highlighted in boldface and italics, respectively. The number of times a given augmentation strategy provides the best or second-best performance for a given descriptor across training set sizes is also reported. The KS distances between the training and the test set molecules and for the designs obtained with no augmentation are reported as a reference (n.a. = not available)

Property	Method	Training set size					Times top-2
		1000	2500	5000	7500	10 000	
HBA	Enumeration	*4 ± 2*	12 ± 1	*2.3 ± 0.7*	7 ± 2	**4.5 ± 0.7**	*3*
	Token deletion (random)	25 ± 11	22 ± 6	22 ± 5	25 ± 8	27 ± 4	0
	Token deletion (validity)	16 ± 2	17 ± 2	13 ± 1	12.9 ± 0.5	20 ± 3	0
	Token deletion (protected)	33 ± 8	14 ± 5	17 ± 5	18 ± 2	17 ± 4	0
	Atom masking (random)	23 ± 2	21 ± 2	13 ± 5	10.8 ± 0.9	7.7 ± 0.7	0
	Atom masking (funct. group)	14 ± 4	**8 ± 3**	10 ± 1	*6 ± 2*	7 ± 2	2
	Bioisosteric substitution	**2.6 ± 0.5**	*10 ± 2*	**2.1 ± 0.7**	**5 ± 2**	*6.8 ± 0.3*	**5**
	Self-training	50.0 ± 0.5	18.0 ± 0.2	14 ± 3	13.0 ± 0.6	13.2 ± 0.9	0
	No augmentation	31 ± 4	16 ± 2	15.4 ± 0.5	18 ± 3	13.4 ± 0.4	0
	Train – test	2	1	1	1	1	n.a.
HBD	Enumeration	*4 ± 3*	**2 ± 1**	**2 ± 2**	**1.8 ± 0.5**	3 ± 1	**4**
	Token deletion (random)	10.3 ± 0.2	8 ± 2	8 ± 2	6 ± 2	10 ± 6	0
	Token deletion (validity)	**4 ± 2**	5 ± 2	*3 ± 1*	4.0 ± 0.5	4.1 ± 0.8	*2*
	Token deletion (protected)	11 ± 4	4 ± 1	4 ± 2	5 ± 2	*2.9 ± 0.1*	1
	Atom masking (random)	**4 ± 2**	5 ± 2	3.7 ± 0.2	*3.2 ± 0.2*	6 ± 2	*2*
	Atom masking (funct. group)	11 ± 3	11 ± 5	4 ± 3	7 ± 3	3.3 ± 0.9	0
	Bioisosteric substitution	5 ± 3	*3 ± 2*	6 ± 3	4 ± 1	**2.2 ± 0.7**	*2*
	Self-training	17 ± 2	4.7 ± 0.9	8 ± 1	14 ± 2	5.7 ± 0.9	0
	No augmentation	14 ± 3	7 ± 1	6 ± 2	4 ± 1	7.2 ± 0.7	0
	Train – test	3	4	2	2	2	n.a.
MW	Enumeration	12.6 ± 0.4	14 ± 2	*8 ± 1*	**5.6 ± 0.6**	*5 ± 1*	*3*
	Token deletion (random)	45 ± 6	31 ± 4	34 ± 7	31 ± 8	32 ± 4	0
	Token deletion (validity)	25.5 ± 0.7	22 ± 3	20 ± 3	20 ± 1	22 ± 2	0
	Token deletion (protected)	43 ± 5	26 ± 3	22 ± 6	28 ± 3	25 ± 4	0
	Atom masking (random)	21 ± 3	21 ± 1	**6 ± 2**	10 ± 5	**4 ± 1**	2
	Atom masking (funct. group)	*11 ± 5*	*9 ± 3*	**6 ± 2**	*6 ± 2*	5 ± 2	**4**
	Bioisosteric substitution	**5.6 ± 1.0**	**8 ± 2**	9 ± 1	7 ± 1	13.1 ± 0.5	2
	Self-training	16.1 ± 0.7	12.1 ± 0.8	11.2 ± 0.9	11 ± 1	7.5 ± 0.1	0
	No augmentation	40 ± 3	21 ± 1	15.3 ± 0.2	17 ± 1	16 ± 2	0
	Train – test	3	3	3	3	3	n.a.
Log *P*	Enumeration	11 ± 3	*7 ± 2*	8 ± 3	**3 ± 1**	**5.1 ± 0.8**	**3**
	Token deletion (random)	31 ± 3	19 ± 4	22 ± 4	18 ± 6	19 ± 2	0
	Token deletion (validity)	17 ± 5	12 ± 1	12 ± 2	13 ± 2	12 ± 3	0
	Token deletion (protected)	32 ± 11	22 ± 4	16 ± 3	22.1 ± 0.6	17 ± 2	0
	Atom masking (random)	11 ± 2	10 ± 1	8 ± 2	7 ± 2	8 ± 2	0
	Atom masking (funct. group)	*8 ± 3*	**6 ± 2**	*4.7 ± 0.7*	7 ± 3	8 ± 2	**3**
	Bioisosteric substitution	**4.8 ± 0.4**	**6 ± 2**	7 ± 4	*4 ± 1*	7.5 ± 0.5	**3**
	Self-training	20 ± 1	7.9 ± 0.5	11 ± 1	11.1 ± 0.8	11 ± 2	0
	No augmentation	14 ± 7	11 ± 2	**3.2 ± 0.7**	12 ± 2	*5.7 ± 0.3*	2
	Train – test	6	5	4	3	3	n.a.

SMILES enumeration is always performed in the top-two approaches across descriptors. When considering the new strategies, atom masking and bioisosteric substitution performed overall the best on distribution learning. This is also visible in the PC analysis (Supporting Fig. S5), showing that enumeration performs best, but atom masking and bioisosteric substitution are close by in performance. Bioisosteric shows the least dependence towards dataset sizes, with bioisosteric substitution ranking consistently among the top two approaches for five out of eight descriptors. Functional group or random masking performs best only in three out of eight properties each, but, in general, shows good results in most properties. Substitution of functional groups can influence certain properties (such as the number of rotatable bonds), but not others – which makes bioisosteric replacement useful for specific goals only (*e.g*., improve selectivity by replacing smaller fragments with bigger ones). Token deletion consistently performed poorly across all properties and sizes for KS values – often even worse than using no augmentation. This is likely due to the detrimental effect of eliminating SMILES tokens on the corresponding molecular properties. Finally, self-training mostly performed slightly worse than not using data augmentation in most cases, with its worst performance for 1000 molecules. This performance trend is expected, since training on smaller datasets (to generate ‘augmented’ SMILES inputs) challenges the distribution learning capabilities of CLMs (Table 1, Supporting. Table S2).

### Effect of augmentation on transfer learning

In low-data scenarios, transfer learning is often utilized rather than training from scratch.^37,38^ Transfer learning allows to ‘pretrain’ a CLM on a large corpora of molecules, and later to fine-tune it on task-specific data (*e.g*., bioactive molecules) to learn the underlying property distribution. To test the potential of the augmentation techniques with transfer learning, we pre-trained a CLM on 1.5 M SMILES strings from ChEMBL.^35^ The pre-trained CLM was then fine-tuned on the molecules tested on three targets,^39^ separately: (1) Peroxisome Proliferator Activated Receptor δ (PPARδ), (2) Serine/threonine-protein kinase (PIM1), and (3) Janus kinase 2 (JAK2). For each target, we created two groups of molecules based on their pairwise substructure similarity (determined as Tanimoto similarity on extended connectivity fingerprints^40^): (1) ‘high-similarity’ molecules, having pairwise similarity larger than or equal to 0.8, and (2) ‘low-similarity’ molecules, whose pairwise similarity was equal to or lower than 0.4. For each of these two similarity scenarios, we created two fine-tuning sets of 10 and 100 molecules. In total, 12 datasets were used for model fine-tuning and molecule generation (1000 SMILES strings sampled across three repetitions) with each augmentation strategy. A 10-fold augmentation was applied to all approaches, whenever possible. If 10-fold augmented SMILES could not be generated (*e.g*., due to a limited number of functional groups to be replaced), augmentation until saturation was performed (Supporting Table S3). Validity, uniqueness, and novelty were monitored for ‘sanity check’^41^ (Supporting Table S4).

**Fig. 3:**
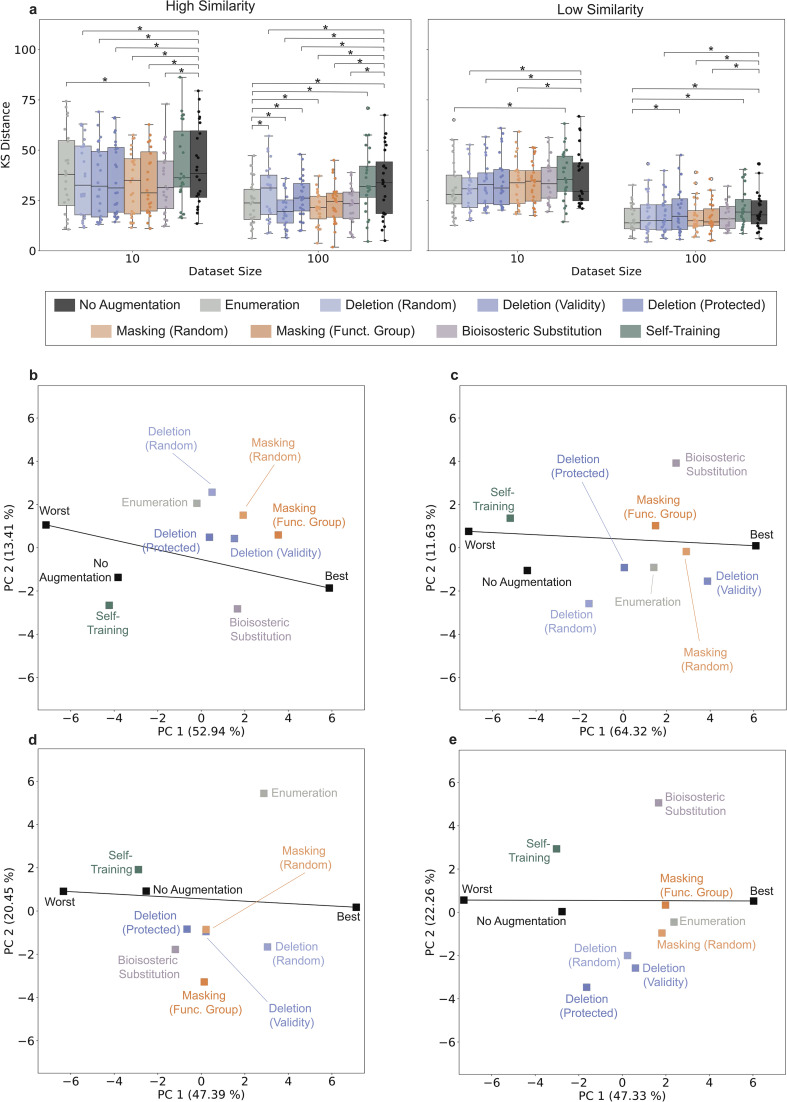
Distribution learning after fine-tuning. The Kolmogorov–Smirnov (KS) distance for eight selected descriptors was calculated between 3000 designs and the respective fine-tuning sets (the lower the KS, the better). (a) KS distances grouped by fine-tuning set similarity (high/low) and number of fine-tuning molecules (10, 100). Statistically significant differences (Wilcoxon signed-rank test, *p* < 0.05) between the new augmentation approaches and no augmentation or SMILES enumeration are marked with asterisks. (b–e) Principal component analysis (PCA) obtained on the KS values for different dataset sizes (b and d: 10; c and e: 100) and similarity levels (b and c: high; d and e: low). ‘Best’ and ‘Worst’ indicate the lowest and highest values of KS obtained across experiments, and the line connecting represents the direction of average performance variation from the best to worst performance.

The methods were analysed for their ability to learn the distribution of the selected molecular properties, measured *via* the Kolmogorov–Smirnov (KS) distance (Supporting Table S5–7). In general, distribution learning is more effective when 100 and/or dissimilar fine-tuning sets are used (Fig. 3a). All augmentation methods performed on a par with SMILES enumeration when 10 highly similar fine-tuning molecules were used. Moreover, functional group masking significantly outperformed SMILES enumeration (Wilcoxon signed-rank test, *p*-value < 0.008). For 100 molecules and highly similar data, we can see that random masking and deletion with enforced validity outperforms SMILES enumeration (*p*-value < 0.03), and functional group masking and bioisosteric substitution perform on a par with SMILES enumeration. In low-similarity scenarios, most methods perform similar to no augmentation (exceptions are enumeration, atom masking, random deletion, and deletion with enforced validity, *p*-value < 0.02) and perform similarly to SMILES enumeration for fine-tuning sets of 10 molecules (Wilcoxon signed-rank test, *α* = 0.05) when general trends are analysed.

To provide a more fine-grained overview of the KS values across descriptors and targets beyond the analysis of general trends, we performed a principal component analysis (PCA). For each dataset size (10 and 100) and similarity level (high, low), the results were described in a tabular form, with each augmentation approach applied to a target being a row, described by 24 KS values (eight descriptors for each targets, across three targets, in comparison to the fine-tuning set) as the columns. As in previous studies,^39,42,43^ to improve interpretability, we added two additional rows: ‘best’ and ‘worst’, corresponding to the minimum and maximum KS values obtained in each column, respectively. This addition ‘stretches’ the variance explained by the first component in the best-worst direction,^39,42,43^ so that the closer a method is to ‘best’ along the best-worst direction, the better it performs on average across descriptors (Fig. 3b–e). Deviations from the best-worst line represent descriptor- and target-dependent variability.^39,42,43^

Except for the scenario with 100 low-similarity fine-tuning data (Fig. 3e), at least one augmentation method outperforms SMILES enumeration on average (Fig. 3b–d). In these cases, random masking or functional group masking are among the best performing methods (and the second and third best in the remaining case, Fig. 3e). The relative performance of the other methods (except for self-training performing consistently poorly) depends on the case study (SI Table S5–7), with no evident trends. These results underscore the potential of atom masking for distribution learning, and the need to investigate the usefulness of the other approaches on a case-by-case basis.

### Molecular scaffold analysis

The analysis of the generated molecular scaffolds holds great importance in drug discovery.^44^ On the one hand, preserving “privileged” molecular scaffolds for bioactivity can serve for molecule optimization,^45^ and, on the other hand, the exploration of structurally distinct compounds having similar activity can accelerate the identification of new therapeutic agents with improved efficacy and selectivity.^46^ For this reason, we used the results of all transfer learning experiments to analyse the generated molecular scaffolds (computed *via* the Bemis-Murcko^47^ algorithm).

**Fig. 4:**
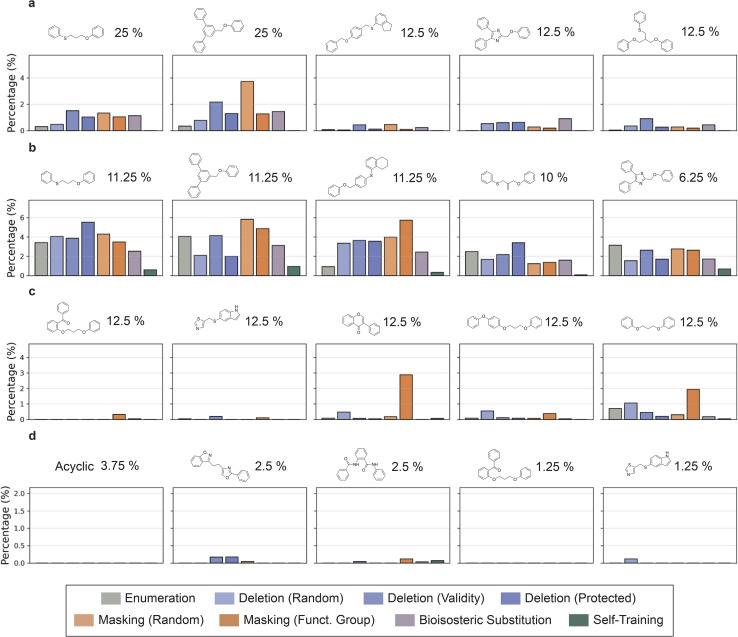
Percentage of the most common scaffolds after training with each method for PPARδ. The most common scaffolds of the PPARδ fine-tuning sets were determined, and for each method, the percentage of the matched scaffold of the 4000 designs was calculated for different dataset sizes (a and c: 10; b and d: 100) and similarity levels (a and b: high; c and d: low). The most common scaffolds are visualized above every graph with the percentage of its occurrence in the fine-tuning set. The analysis for PIM and JAK2 can be found in Supporting Fig. S6 and S7, respectively.

First, we analysed the five most frequent scaffolds and compared them with the five most frequent scaffolds in the respective fine-tuning sets (Fig. 4 [PPARδ], and Supporting Fig. S6 and 7 [PIM1, JAK2]). In general, using more similar molecules (Fig. 4a and b) for fine-tuning leads to a better matching of the most frequent molecular scaffolds by the CLMs. In such high-similarity settings, most methods (except for self-training) have a similar or better ability to reproduce ‘recurrent’ scaffolds than SMILES enumeration (Fig. 4a and b). This observation suggests a better capability to learn the underlying structural features of the fine-tuning sets compared to SMILES enumeration. Atom masking showed the best ability to reproduce frequent scaffolds across experiments for both high and low similarity datasets, followed closely by token deletion.

Another desirable property when performing *de novo* design is the capacity to generate chemically diverse structures that go beyond the molecules used for training. To this end, we analysed the ability of each augmentation strategy to generate diverse and novel molecular scaffolds^47^ compared to the molecules used for training. Using all the molecules generated during the transfer learning experiments, we measured (a) scaffold diversity, *i.e.*, the number of novel scaffolds within the sampled molecules, and (b) scaffold novelty, *i.e.*, the number of sampled scaffolds that are not in the fine-tuning or pre-training sets. The values obtained without using augmentation were reported as a baseline.

Performing no data augmentation yields usually high or best results in the creation of diverse and novel scaffolds. In all cases, at least two augmentation strategies perform better than SMILES enumeration when it comes to generating diverse and novel molecular scaffolds for very low-data settings (Table 2). Token deletion performs on average the best, regardless of the molecular similarity, the number of fine-tuning molecules and the macromolecular target. These results are owing to the nature of the approach, which perturbs the input by generating diverse SMILES for training (3–70% scaffold novelty in the training set, Supporting Table S8). The other methods based on input ‘perturbation’ (bioisosteric substitution, self-training, Supporting Table S8) also often show top performances across targets. In general, the performance of the other augmentation methods in comparison with SMILES enumeration depends on the considered target and fine-tuning scenario.

**Table 2:** Scaffold diversity and novelty. Metrics were measured after fine-tuning on bioactive molecules for three targets (PPAR, PIM1, and JAK2) using 10 and 100 molecules selected with (a) high similarity and (b) low similarity. Scaffold diversity and novelty relative to the fine-tuning sets (FT) and pre-training sets (PT) are reported as the mean ± standard deviation for 10 fine-tuning molecules. For each experimental setup and each metric, the best and second best values are reported in boldface and italics, respectively

Similarity	Target	Augmentation	10 fine-tuning molecules			100 fine-tuning molecules		
			Scaffold diversity	Scaffold novelty (FT)	Scaffold novelty (PT)	Scaffold diversity	Scaffold novelty (FT)	Scaffold novelty (PT)
High	PPARδ	Enumeration	67 ± 2	67 ± 2	35 ± 1	60 ± 1	56 ± 1	*42 ± 1*
		Token deletion (random)	78 ± 1	78 ± 1	46 ± 1	*70 ± 2*	*62 ± 2*	41.8 ± 0.9
		Token deletion (validity)	77.0 ± 0.9	76.3 ± 0.9	45 ± 1	52 ± 2	48 ± 2	35 ± 2
		Token deletion (protected)	82 ± 1	81 ± 1	46 ± 3	64.5 ± 0.8	56.3 ± 0.8	37 ± 3
		Atom masking (random)	77 ± 3	75 ± 2	44 ± 1	61.3 ± 0.8	53 ± 1	35 ± 2
		Atom masking (funct. Group)	81 ± 1	80 ± 1	45 ± 1	63 ± 4	55 ± 4	35 ± 2
		Bioisosteric substitution	80 ± 1	80 ± 1	*51 ± 3*	54 ± 2	51 ± 2	35.2 ± 0.3
		Self-training	*83 ± 1*	*83 ± 1*	40.1 ± 0.9	55.3 ± 0.9	53.8 ± 0.9	25 ± 1
		No augmentation	**93 ± 1**	**93 ± 1**	**52 ± 3**	**77 ± 1**	**75 ± 1**	**44 ± 1**
	PIM1	Enumeration	93.6 ± 0.3	93.2 ± 0.2	58.2 ± 0.8	91 ± 2	*88 ± 2*	**77 ± 1**
		Token deletion (random)	94.1 ± 0.1	93.6 ± 0.1	*59.9 ± 0.1*	*92 ± 2*	85 ± 2	*75 ± 2*
		Token deletion (validity)	93.4 ± 0.9	92.9 ± 1.0	57 ± 2	83 ± 2	77 ± 2	71 ± 2
		Token deletion (protected)	*94.4 ± 0.2*	*93.9 ± 0.1*	**60 ± 2**	*92 ± 2*	86 ± 2	74.5 ± 0.8
		Atom masking (random)	90.5 ± 0.4	89.8 ± 0.5	56 ± 2	77.1 ± 0.9	68.3 ± 0.4	57 ± 2
		Atom masking (funct. Group)	86.1 ± 0.4	85.3 ± 0.4	51 ± 1	80.1 ± 0.6	72.6 ± 0.9	55 ± 2
		Bioisosteric substitution	92.8 ± 0.8	92.5 ± 0.8	53.5 ± 1.0	83 ± 2	81 ± 2	56.7 ± 0.5
		Self-training	86.5 ± 0.8	86.5 ± 0.8	45 ± 1	85.7 ± 0.4	84.0 ± 0.5	48 ± 1
		No augmentation	**94.7 ± 0.7**	**94.5 ± 0.7**	54.6 ± 0.6	**93 ± 2**	**91 ± 1**	56.5 ± 0.7
	JAK2	Enumeration	93.2 ± 0.4	93.2 ± 0.4	63.8 ± 0.2	87.1 ± 0.8	85 ± 1	**73 ± 2**
		Token deletion (random)	*96.8 ± 0.9*	*96.3 ± 0.9*	70 ± 2	*91 ± 1*	*87.2 ± 0.4*	*72 ± 3*
		Token deletion (validity)	95.8 ± 0.5	95.1 ± 0.4	*75 ± 2*	78.2 ± 0.7	76.3 ± 0.7	68 ± 1
		Token deletion (protected)	**97.3 ± 0.4**	**96.7 ± 0.4**	**76.1 ± 0.6**	89 ± 2	85 ± 2	72 ± 4
		Atom masking (random)	93 ± 1	92 ± 1	68 ± 1	76.8 ± 0.7	72 ± 1	55.5 ± 0.8
		Atom masking (funct. Group)	93.8 ± 0.6	92.7 ± 0.7	68 ± 2	81.0 ± 0.3	77.7 ± 0.9	60 ± 2
		Bioisosteric substitution	92.8 ± 0.2	92.0 ± 0.2	67.5 ± 0.2	75.1 ± 1.0	71.4 ± 1.0	59 ± 1
		Self-training	87 ± 1	87 ± 1	49 ± 2	86.0 ± 0.4	85.2 ± 0.3	51 ± 1
		No augmentation	94.8 ± 0.4	94.8 ± 0.4	58.4 ± 0.3	**93 ± 1**	**91 ± 1**	61 ± 1
Low	PPARδ	Enumeration	76.9 ± 0.3	76.6 ± 0.3	40.1 ± 0.8	86.1 ± 0.6	84.7 ± 0.8	**58 ± 2**
		Token deletion (random)	85.6 ± 1.0	85.0 ± 1.0	47 ± 2	81.2 ± 0.7	77 ± 1	42 ± 1
		Token deletion (validity)	91.0 ± 0.9	90.5 ± 1.0	48 ± 3	84.5 ± 0.5	82.4 ± 0.4	46.1 ± 0.9
		Token deletion (protected)	*91.5 ± 0.6*	*91.3 ± 0.6*	*52 ± 2*	86 ± 1	84 ± 1	46 ± 1
		Atom masking (random)	90.0 ± 0.6	89.7 ± 0.6	49 ± 1	83 ± 3	80 ± 2	42 ± 2
		Atom masking (funct. Group)	83 ± 2	82 ± 2	44 ± 2	82.3 ± 0.8	79.4 ± 0.9	44 ± 1
		Bioisosteric substitution	90.0 ± 1.0	89.7 ± 0.9	51 ± 2	**91 ± 1**	**89 ± 1**	*55.0 ± 0.8*
		Self-training	89 ± 1	89 ± 1	46.2 ± 0.4	71 ± 1	71 ± 1	33 ± 1
		No augmentation	**94 ± 2**	**94 ± 2**	**54 ± 2**	*88.9 ± 0.8*	*88.0 ± 0.9*	46 ± 3
	PIM1	Enumeration	90.0 ± 0.3	89.8 ± 0.2	47.6 ± 0.7	93.5 ± 0.3	91.6 ± 0.5	**63 ± 1**
		Token deletion (random)	94.6 ± 0.6	93.9 ± 0.6	55.6 ± 0.7	92.6 ± 0.1	90.0 ± 0.4	54.0 ± 1.0
		Token deletion (validity)	94.9 ± 0.2	94.8 ± 0.2	*56 ± 1*	**94.9 ± 0.5**	93.0 ± 0.7	55 ± 2
		Token deletion (protected)	*95.5 ± 0.4*	*95.4 ± 0.4*	**57.4 ± 0.5**	*94.5 ± 0.4*	92.5 ± 0.7	*55 ± 1*
		Atom masking (random)	**96.0 ± 0.4**	**95.6 ± 0.3**	54 ± 2	93.9 ± 0.3	91.8 ± 0.7	52.4 ± 0.9
		Atom masking (funct. Group)	94.7 ± 0.3	94.2 ± 0.3	55 ± 1	91.5 ± 0.3	88.3 ± 0.2	49.6 ± 0.4
		Bioisosteric substitution	95.2 ± 0.6	95.0 ± 0.5	55 ± 2	94.4 ± 0.6	*93.4 ± 0.6*	*55 ± 1*
		Self-training	88.6 ± 0.5	88.6 ± 0.5	46.5 ± 0.9	87.9 ± 0.9	87.5 ± 0.8	42.2 ± 0.4
		No augmentation	94.8 ± 0.9	94.7 ± 0.8	54 ± 2	94.5 ± 0.7	**93.8 ± 0.6**	53 ± 2
	JAK2	Enumeration	83 ± 1	83 ± 1	39.8 ± 0.3	94.3 ± 0.9	93 ± 1	**66 ± 1**
		Token deletion (random)	91.9 ± 0.5	91.3 ± 0.4	51 ± 1	95 ± 1	93 ± 2	*59 ± 1*
		Token deletion (validity)	93.1 ± 0.6	92.8 ± 0.6	51.8 ± 0.4	95.0 ± 0.8	93.5 ± 1.0	56 ± 2
		Token deletion (protected)	91.3 ± 0.7	90.6 ± 0.6	51 ± 1	**96.7 ± 0.9**	**96 ± 1**	*59 ± 1*
		Atom masking (random)	90.8 ± 0.4	90.3 ± 0.4	52.3 ± 0.9	93.5 ± 0.4	91.1 ± 0.5	55 ± 2
		Atom masking (funct. Group)	92.9 ± 0.3	92.4 ± 0.2	*54 ± 2*	94.1 ± 0.2	92.2 ± 0.5	56 ± 2
		Bioisosteric substitution	**94.5 ± 0.2**	**94.3 ± 0.1**	**54.3 ± 0.9**	94.7 ± 0.6	94.2 ± 0.7	57.5 ± 0.9
		Self-training	87.6 ± 0.7	87.5 ± 0.6	44.8 ± 0.7	87 ± 1	86.6 ± 0.9	41.4 ± 0.9
		No augmentation	*94 ± 1*	*94 ± 1*	53 ± 1	*96 ± 1*	**96 ± 1**	53.3 ± 0.3

By combining these two facets of scaffold analysis, token deletion results in the most promising approach for exploring both novel chemical scaffolds and decorations of recurring scaffolds. Atom masking – while still producing good values of novelty and diversity – is better suited to decorating recurring fine-tuning scaffolds). Like enumeration, bioisosteric substitution is a valuable option for both scaffold decoration and scaffold exploration, with a dataset-dependent performance. These results confirm the value of optimizing the chosen SMILES augmentation strategies when utilizing generative deep learning for chemical space exploration and/or molecule optimization.

## Conclusions and outlook

In chemical language modelling, SMILES enumeration has showed incredible results for data augmentation. In this work, we rethink how SMILES strings can be augmented for *de novo* design with chemical language models. In particular, we introduced four augmentation strategies (and several variants) and systematically analysed their ability to generate molecules with desirable properties and relevant molecular scaffolds. This systematic study shed light on the different advantages and unique features of each augmentation strategy. While this study has relied only on LSTMs, the augmentation strategies reported herein can be applied in principle to any neural network architecture suited for sequences.

Our study reveals that some of these methods can advance chemical language modelling further in comparison with the well-established SMILES enumeration. No augmentation strategy is able to ‘rule them all’, but the optimal approach depends on the overall goal. When training from scratch with small datasets (*e.g*., less than 5000 training molecules), different augmentation methods allow matching different physico-chemical properties differently. In this context, bioisosteric replacement, self-training and atom masking are particularly interesting alternatives to SMILES enumeration, depending on the property of interest. When combined with transfer learning, atom masking and deletion with enforced validity confirmed their potential to perform similar to or better than SMILES enumeration in their distribution learning and scaffold matching capabilities, especially with (a) low-data regimes (*i.e.*, 10 fine-tuning molecules) or (b) fine-tuning sets composed of highly similar molecules. The other augmentation strategies showed a task-dependent performance. When it comes to navigating the chemical space in search for diverse molecules, strategies that perturb the input SMILES for augmentation (*e.g*., token deletion and bioisosteric substitution) show the highest potential to provide novel scaffolds (while still managing to match scaffolds from the training set). These results underscore the opportunities of these new augmentation strategies to further accelerate experimental *de novo* design campaign. We expect each one of these techniques to be better suited for chemical space exploration (*e.g*., bioisosteric replacement and token deletion) or library enlargement (*e.g*., atom masking). In future works, the combination of different augmentation strategies presents a promising direction, which could further improve the results.

While our study only focused on SMILES strings, its results can be applied to virtually any molecular line notation such as (Group)SELFIES,^12,48^ fragSMILES^12,48,49^ and SAFE.^50^ Moreover, while here we focused on distribution learning, these newly introduced augmentation techniques are expected to support other learning regimes, such as reinforcement learning.^51^ In this context, we expect approaches that allow for a higher diversity of molecular designs (*e.g*., token deletion and bioisosteric replacement) to be particularly beneficial to explore uncharted regions in the chemical space, steered by model rewards. Finally, the approaches presented herein are easy to expand based on the user needs (*e.g*., by specifying a different set of functional groups to be considered/replaced for masking and bioisosteric substitution) and are hence expected to show additional potential in the future. While some of the newly introduced augmentation strategies are beneficial to increase the quality of the *de novo* designs, their suitability to other molecular tasks (*e.g*., structure–activity or structure–property relationship prediction) has yet to be demonstrated by additional studies.

## Materials and methods

### Data collection and curation

#### ChEMBL data collection and preprocessing

2 372 647 molecules in the form of SMILES strings were collected from the ChEMBL^35^ database (v. 33). Salts and corresponding charges were removed, stereochemistry information was eliminated, and SMILES strings were sanitized. Duplicates were removed, and SMILES strings that contained atoms different than a predefined set (corresponding to the tokens ‘C’, ‘O’, ‘N’, ‘S’, ‘P’, ‘F’, ‘Cl’, ‘Br’, ‘I’, ‘c’, ‘n’, ‘o’, and ‘s’) were eliminated. Canonical SMILES strings shorter than six and longer than 150 tokens were eliminated. Lastly, a randomized SMILES string was created for each molecule.

#### Dataset creation for training size analysis

From ChEMBL, we created several subsets to investigate the effect of the training data size. Here, 50 000 SMILES strings were randomly sampled for follow-up clustering. A spectral clustering algorithm^52^ was used to cluster the SMILES strings based on their generic Bemis-Murcko^47^ scaffolds. Stratified sampling by cluster assignation on 25 000 SMILES strings was used to create the datasets of different sizes (10 000, 7500, 5000, 2500, and 1000) and ensure that smaller datasets were included in the bigger ones for comparability. Each dataset was randomly divided into a training (90%) and a validation (10%) set. From the remaining 25 000 SMILES strings, a test set (1000 SMILES strings) was obtained *via* cluster-based stratification. The SMILES strings were then tokenized,^16^ and the start-of-the-sequence (‘G’) and end-of-the-sequence tokens (‘E’) were added.^53^ The tokenized SMILES strings were padded to the maximum length (150 tokens) and one-hot encoded.

#### Transfer learning data

(1) Pre-training. The curated ChEMBL dataset was used (2 213 855 molecules) for further curation. A single, randomized, SMILES string was used for pre-training, to not (dis)favour any augmentation technique. The dataset was randomly divided into a training (70%, 1 549 696 molecules), a validation (10%, 221 385 molecules), and a test (20%, 442 771 molecules) set. (2) Fine-tuning. Three macromolecular targets were chosen from the MoleculeACE^54^ repository: Peroxisome Proliferator Activated Receptor-δ (PPARδ), Serine/threonine-protein kinase (PIM1), and Janus kinase 2 (JAK2). These datasets were pre-processed as mentioned before. Afterwards, similar and dissimilar sets of two different sizes (10, 100) were created. Datasets of similar molecules were created by performing agglomerative clustering, as reported previously.^55^ To reach high similarity, 20 parent clusters and 40 subclusters among the parent clusters were determined. Afterwards, the clusters and subclusters having more than the target number of molecules (10 or 100) were analysed for their Tanimoto similarity on Extended Connectivity Fingerprints (ECFPs, length = 1024 bits, radius = 2 bonds). Molecules with high pairwise similarity with each other (larger than or equal to 0.8) were assigned to the fine-tuning set of highly similar molecules. To obtain low-similarity datasets, we used the function Leader_Picker of RDKit to identify molecules with a Tanimoto similarity lower than or equal to 0.4.

### Data augmentation

SMILES enumeration was performed as proposed previously.^15^ For the other strategies, augmentation was performed as follows:

#### • Token deletion

Token deletion took place after vocabulary creation and tokenization. Each token of a molecule was parsed and deleted with a probability *p*. Validity was enforced by sanitizing the token-depleted SMILES strings and discarding the invalid SMILES strings. In protected deletion, the removal of tokens identifying ring structures (numbers from ‘1’ to ‘9’, and ‘%’), and branches (‘(‘ and ’)’) was not allowed.

#### • Atom masking

After transforming the SMILES strings into an RDKit molecular object, each atom within the molecule was masked with a probability *p* using the dummy atom ‘*‘. Each atom in the molecular object was parsed and replaced. For functional group masking, SMARTS patterns were used to identify substructures to mask. A test was conducted to ensure that the masked and original SMILES string only differ in the ‘*’ token. Only parts of the SMILES input to the model are masked; the target remains the original, unmasked SMILES string.

#### • Bioisosteric substitution

Molecules were fragmented using the ‘Breaking of Retrosynthetically Interesting Chemical Substructures’ (BRICS) algorithm.^56^ The list of possible replacements for each substructure was retrieved from SwissBioisostere.^57^ SwissBioisostere, along with the possible replacements, also include the frequency of how many a certain bioisosteric replacement was found to occur (based on better, similar or worse performance in bioactivity). The top five most frequent replacements were chosen as candidates for augmentation. Each molecule was parsed for ‘augmentable’ fragments, the matching fragments were substituted with a probability *p* with one of the candidate fragments, and the molecule was then re-assembled and converted into a SMILES string.

#### • Self-training

After hyperparameter optimization (as described below), the CLMs were trained with all available, non-augmented training SMILES strings (in their non-canonical version), using temperature sampling^17^ (*T* = 0.5, eqn (1)). The trained CLMs were used to generate *de novo* designs. Valid, novel, and unique SMILES strings were retained and used to augment the training set (with the selected augmentation fold).

In this work, an *n*-fold augmentation of a molecule refers to using the original SMILES string along with (*n-1*) additional SMILES strings generated *via* a chosen augmentation approach. All procedures were applied to achieve the desired or highest possible augmentation fold, and with the desired probability of perturbation (*p*) for token deletion, atom masking and bioisosteric substitution (*p* = 0.05, 0.15, 0.30). All augmentation methods were checked for uniqueness and for their presence in the original training dataset.

### Model optimization and training

For each augmentation method, the same model architecture, loss, and hyperparameters were used.

#### Model training, and hyperparameter optimization

Recurrent neural networks with long short-term memory (LSTM, unidirectional) were optimized using hyperparameter values in agreement with the literature:^10,17^ (a) number of LSTM layers = [2, 3], (b) number of hidden units of the LSTM layer = [256, 512], (c) learning rate = [0.001, 0.005, 0.0001]; (d) batch size = [32, 64, 128]. Softmax activation and Adam optimizer were used. Each combination was trained for 500 epochs, and early stopping on the cross-entropy loss in validation was applied (patience = 10, minimum loss change = 0.0001). The model with the best validation loss was used to sample 1000 SMILES strings with a sampling temperature^17^ of *T* = 1.0 (multinomial sampling, eqn (1)), across three independent repeats.

#### Transfer learning

Hyperparameters were chosen in agreement with the literature.^10,17^ For pre-training, three LSTM layers with 512 hidden units each, a learning rate of 0.0005, and a batch size of 512 were chosen, in combination with softmax activation and Adam optimizer. The model was pre-trained for 500 epochs, and early stopping on the cross-entropy loss in validation was applied (patience = 10, minimum loss change = 0.0001). The trained model was used to sample 1000 SMILES strings across three repeats with multinomial sampling^17^ (*T* = 1.0, eqn (1)). During fine-tuning, a learning rate of 0.0000005 and a clipping norm of 1 were used. The model was fine-tuned for 500 epochs with early stopping and sampling as for the pre-training.

### Molecule generation and evaluation

#### Temperature sampling

Molecules were generated *via* temperature sampling, which controls the randomness of the generation. In particular, given a trained CLM, the probability of sampling the *i*-th token of the vocabulary at a given portion of a SMILES string is determined as follows:1
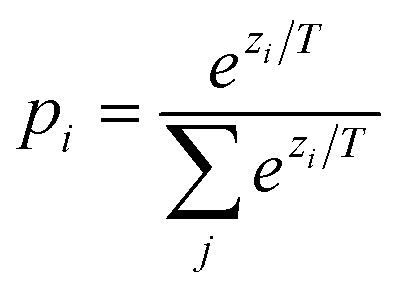
where *z*_*i*_ is the CLM (logit) output for the *i*-th token, and *j* runs over all SMILES tokens in the vocabulary. The temperature value (*T*) controls the randomness of the sampling: *T* = 1 corresponds to standard softmax sampling with no post-hoc modification of the probabilities (multinomial sampling), *T* > 1 allows generating more diverse outputs, while *T* < 1 promotes higher-probability tokens, resulting in more deterministic and repetitive outputs. In this work, we used *T* = 1.0 (multinomial sampling) for CLM evaluation. For self-training augmentation, a value of *T* = 0.5 was used.

#### Evaluation

The sampled SMILES strings were evaluated for their validity, uniqueness, and novelty using tools available in the RDKit. Eight molecular descriptors were computed: number of aliphatic and aromatic rings, molecular weight, partition coefficient (log *P*), number of hydrogen bond acceptors and donors, number of rotatable bonds, and topological surface area (TPSA). The Kolmogorov–Smirnov (KS) distance was computed as implemented in scipy (scipy.kstest). Scaffold diversity and novelty^53^ were calculated by determining their Bemis-Murcko^47^ scaffold of each valid molecule.

## Software and code

All calculations were performed in a Python (v. 3.9.18) environment. We used RDKit v. 2023.9.5 (ref. 58) for molecule handling, SMILES canonicalization, processing and sanification, and for the calculation of molecular fingerprints, scaffolds and descriptors. Clustering was performed with scikit-learn (v. 1.3.0), scipy (v. 1.13.1) and kneed (v. 0.8.5). CLMs were trained using Keras (v. 3.4.1) with a Tensforflow (v. 2.17.0) back-end. ChatGPT (version GPT-4, 2025) assisted in the generation of the graphical abstract.

## Author contributions

Conceptualization: H. B. and F. G. data curation: H. B. formal analysis: H. B. with contributions from A. A. and H. t. S. methodology: all authors. Investigation: all authors. Software: H. B., A. A., H. t. S. visualization: H. B. writing – original draft: H. B. writing – review and editing: H. B. and F. G., with contributions from all authors. All the authors have given approval to the final version of the manuscript.

## Conflicts of interest

There are no conflicts to declare.

## Supplementary Material

DD-004-D5DD00028A-s001

## Data Availability

The datasets and the Python code to replicate and extend our study are freely available on GitHub at the following URL: https://github.com/molML/fantasticSMILESaugmentation. The code and the data at the time of publishing are available on Zenodo: https://doi.org/10.5281/zenodo.16538381. Supplementary information: Fig. S1–S7, and Tables S1–S8. See DOI: https://doi.org/10.1039/d5dd00028a.
