# Supplementary material for: Going beyond SMILES enumeration for data augmentation in generative drug discovery
Source: Digit Discov. 2025 Aug 14;4(10):2752–64. doi: 10.1039/d5dd00028a (PMC12409607; doi:10.1039/d5dd00028a)
Supplement: DD-004-D5DD00028A-s001 [file DD-004-D5DD00028A-s001.pdf]

Supplementary Information (SI) for Digital Discovery.
This journal is © The Royal Society of Chemistry 2025

Going beyond SMILES enumeration for data augmentation in generative drug discovery

Helena Brinkmann, Antoine Argante, Hugo ter Steege, Francesca Grisoni

Supporting Figures

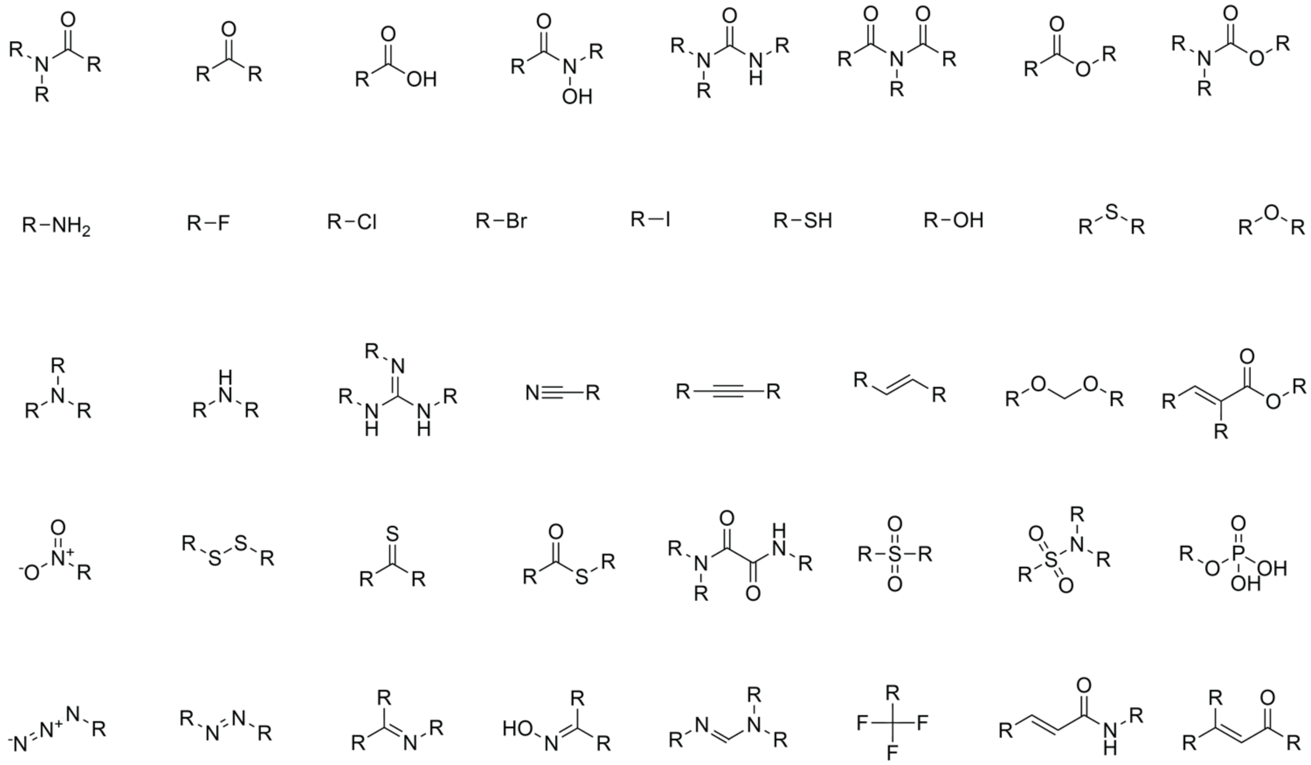

Supporting Figure S1. Functional groups used for functional-group masking or bioisosteric substitution with bioisosters. The functional groups were selected from Ertl *et al.*[1].

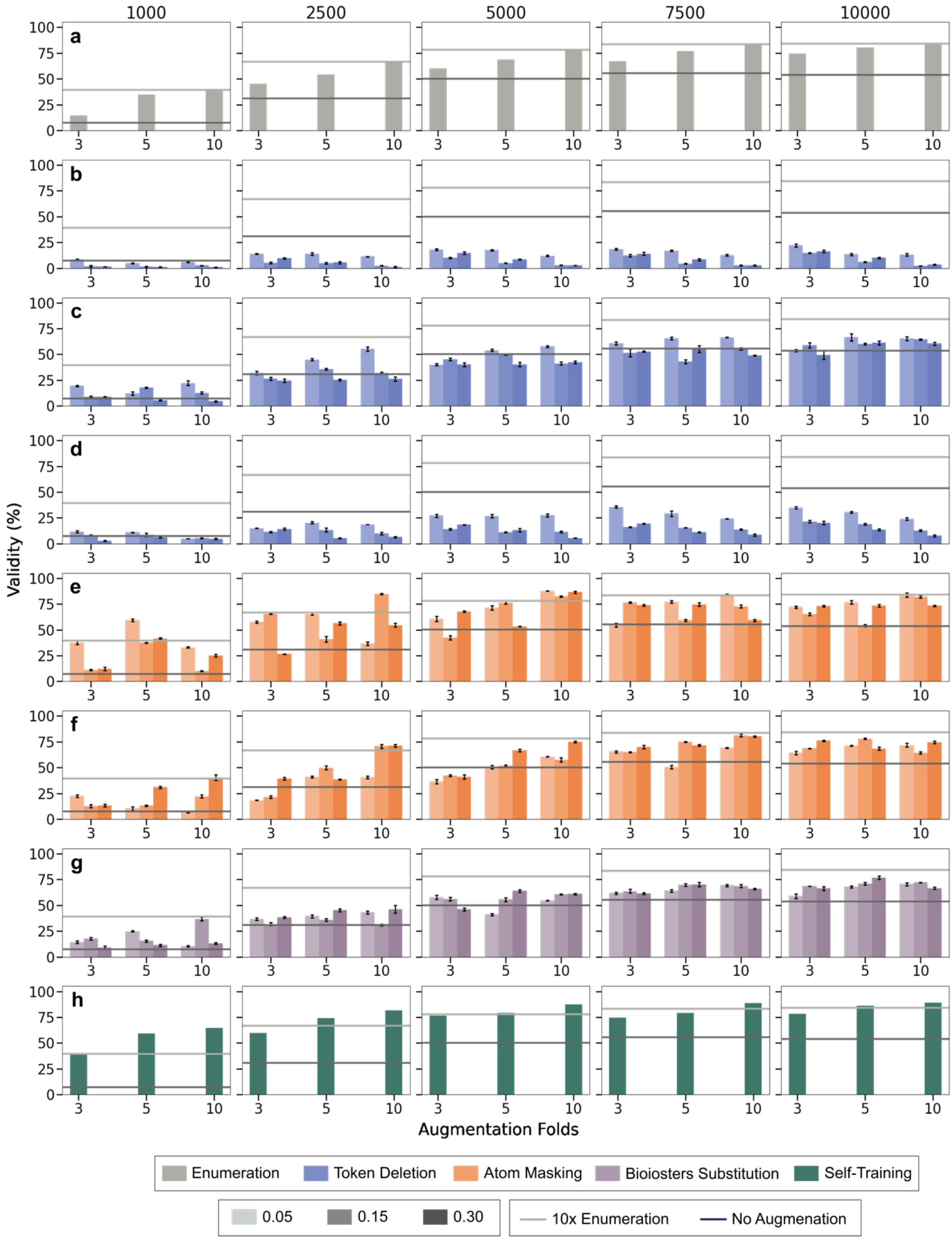

Supporting Figure S2. Syntactic validity of SMILES across augmentation strategies, folds, perturbation probabilities and dataset sizes. Several folds of augmentation (three-, five- and ten-folds), three perturbation probabilities (p = 0.05, p = 0.15 and p = 0.30) and six training set sizes (1000, 2500, 5000, 75000, and 10000 SMILES) were analyzed. For each set-up, 1000 SMILES strings were generated across four repetitions for the analysis. The highest validity obtained by SMILES enumeration and validity obtained with no augmentation for each set-up is reported as a solid line across all experiments for comparison.

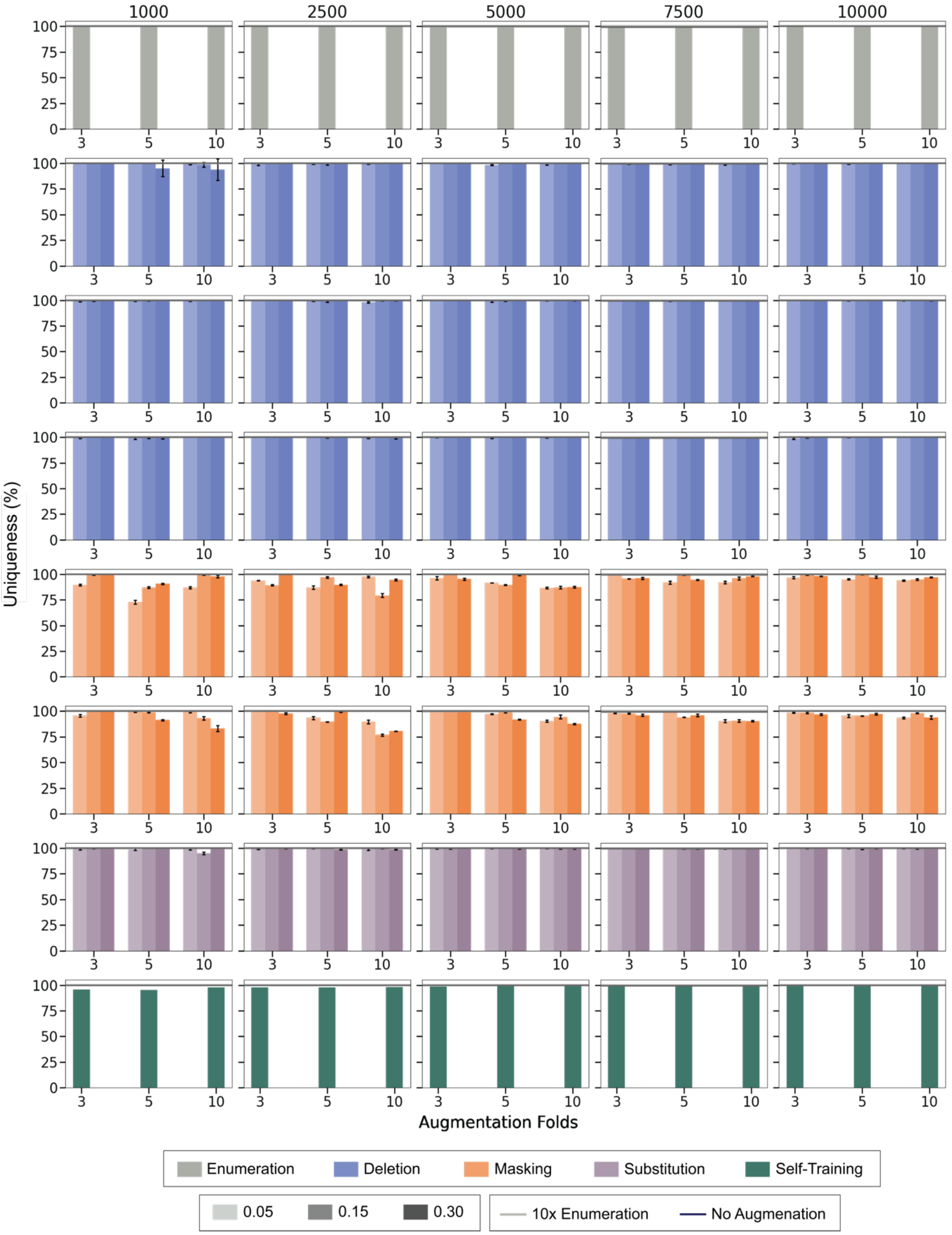

Supporting Figure S3. Uniqueness of valid SMILES strings across augmentation strategies, folds, perturbation probabilities and dataset sizes. Several folds of augmentation (three-, five- and ten-folds), three perturbation probabilities (p = 0.05, p = 0.15 and p = 0.30) and six training set sizes (1000, 2500, 5000, 75000, and 10000 SMILES) were analyzed. For each set-up, 1000 SMILES strings were generated across three repetitions for the analysis. The highest uniqueness obtained by SMILES enumeration for each set-up and uniqueness obtained with no augmentation is reported as a solid line across all experiments for comparison.

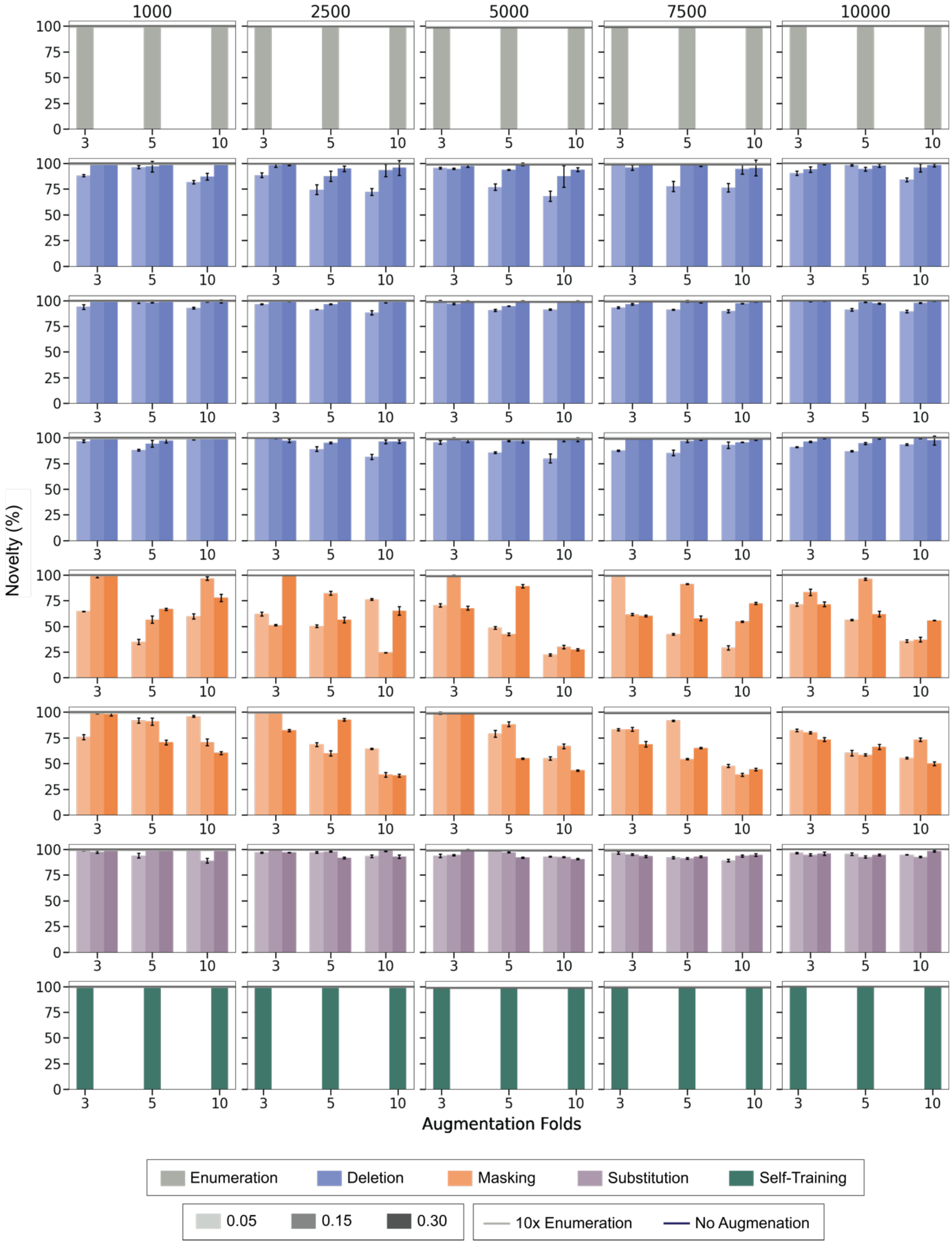

Supporting Figure S4. Novelty of valid SMILES strings across augmentation strategies, folds, perturbation probabilities and dataset sizes. Several folds of augmentation (three-, five- and ten-folds), three perturbation probabilities (p = 0.05, p = 0.15 and p = 0.30) and six training set sizes (1000, 2500, 5000, 75000, and 10000 SMILES) were analyzed. For each set-up, 1000 SMILES strings were generated across three repetitions for the analysis. The highest novelty obtained by SMILES enumeration for each set-up and uniqueness obtained with no augmentation is reported as a solid line across all experiments for comparison.

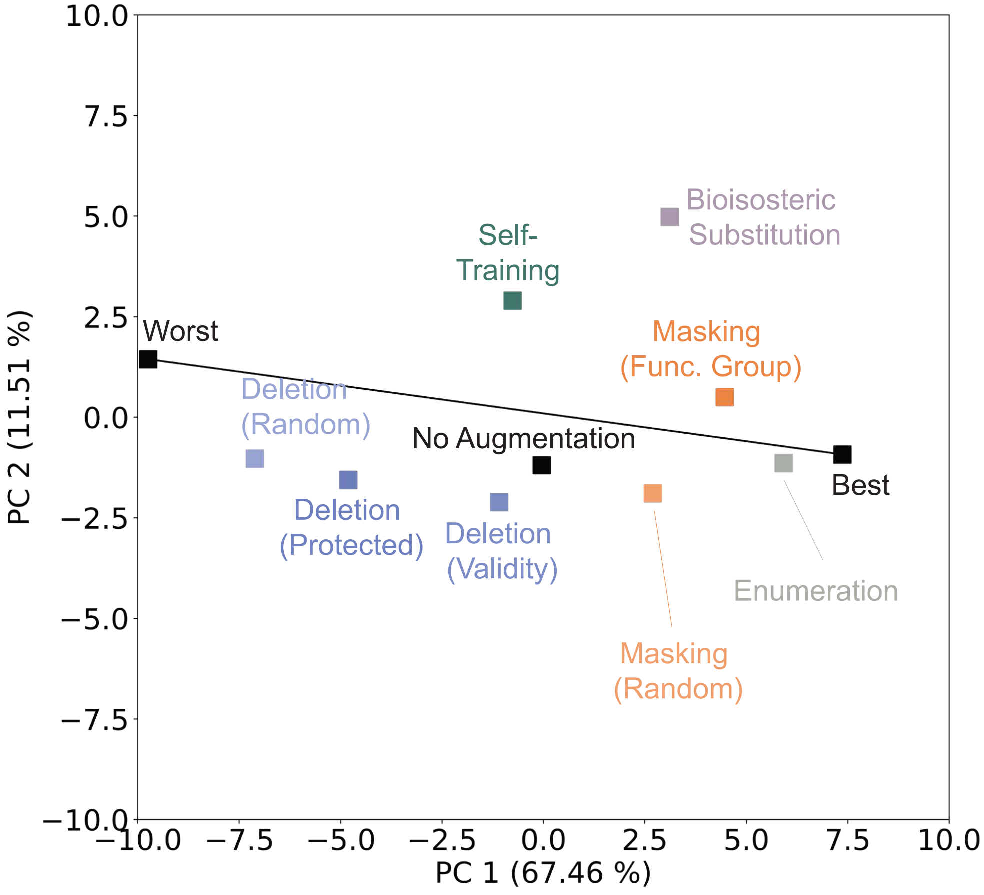

Supporting Figure 5. Principal component analysis (PCA) obtained on the KS values for different dataset sizes and properties. 'Best' and 'Worst' indicate the lowest and highest values of KS obtained across experiments, and the line connecting represents the direction of average performance variation from best to worst performance.

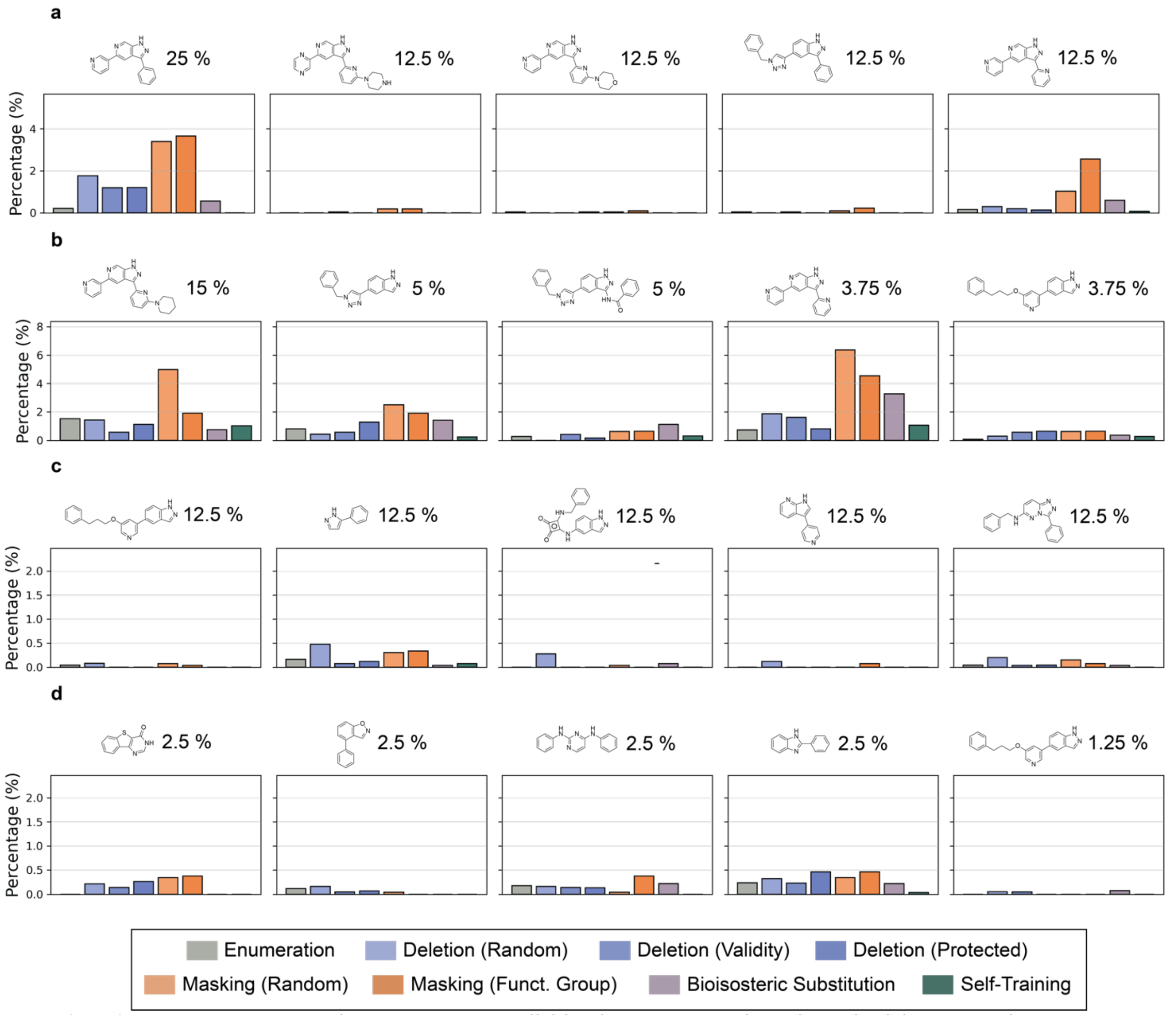

Supporting Figure S6. Percentage of most common scaffolds after training with each method for PIM1. The most common scaffolds of the PIM1 fine-tuning sets were determined and for each method, the percentage of matched scaffold of the 4000 designs were calculated for different dataset sizes (**a**, **c**: 10; **b**, **d**: 100) and similarity levels (**a**, **b**: high; **c**, **d**: low). The most common scaffolds are visualized above every graph with the percentage of its occurrence in the fine-tuning set.

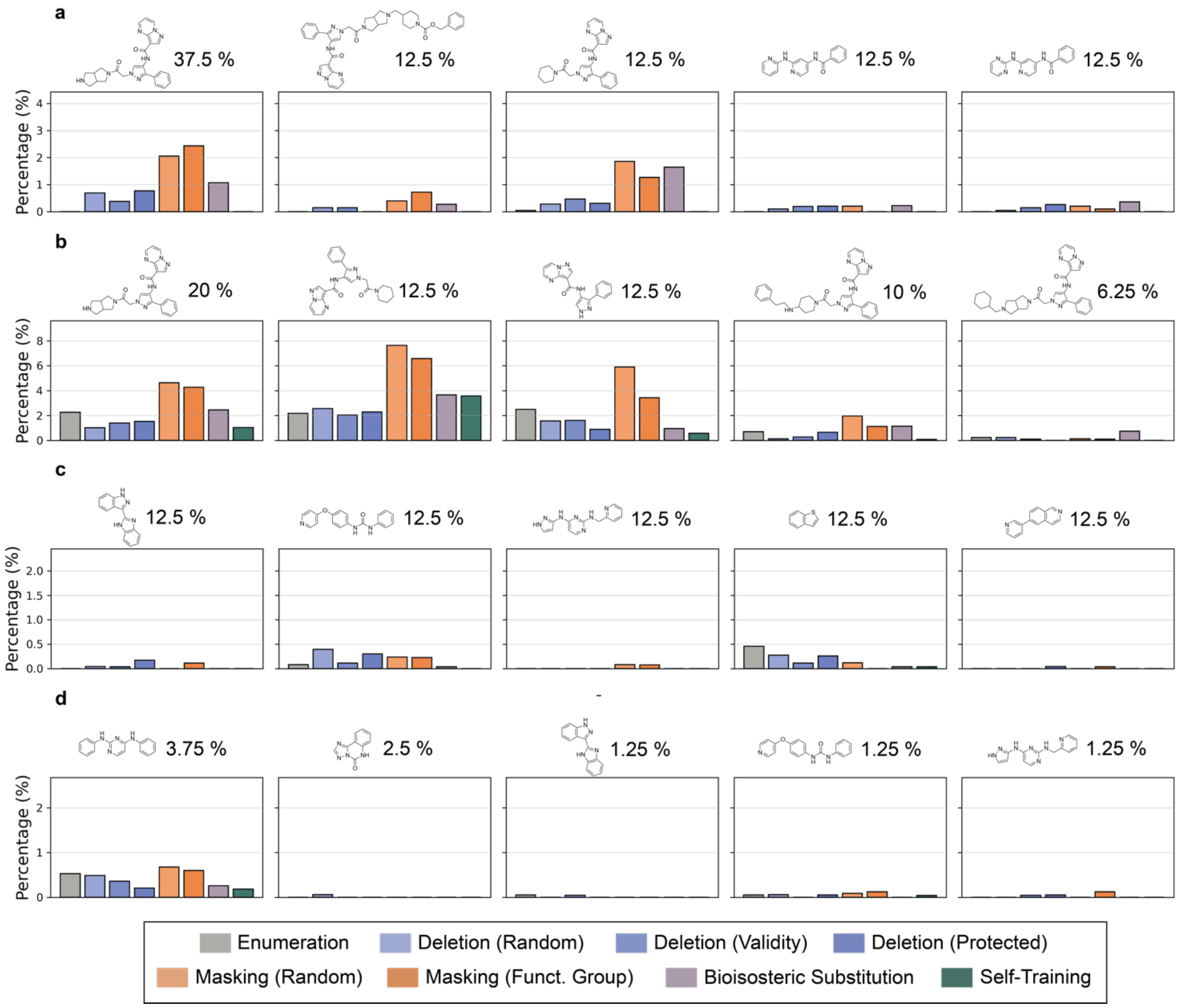

Supporting Figure S7. Percentage of most common scaffolds after training with each method for JAK2. The most common scaffolds of the JAK2 fine-tuning sets were determined and for each method, the percentage of matched scaffold of the 4000 designs were calculated for different dataset sizes (**a**, **c**: 10; **b**, **d**: 100) and similarity levels (**a**, **b**: high; **c**, **d**: low). The most common scaffolds are visualized above every graph with the percentage of its occurrence in the fine-tuning set.

Supporting Tables

Supporting Table S1. Average achieved fold of a SMILES string by augmentation method and all probabilities. We report the achieved fold of each augmentation method compared to the wanted augmentation fold for each method and dataset size for the general analysis of different dataset sizes.

Proba-bility	Size	Fold	Deletion (Random)	Deletion (Validity)	Deletion (Protected)	Masking (Random)	Masking (Func. Group)	Bioisosteric Replace-ment
**p = 0.05**	1000	3	3 ± 0	2.93 ± 0.33	3 ± 0	2.23 ± 0.89	3 ± 0	2.1 ± 0.96
		5	5 ± 0	4.73 ± 0.82	5 ± 0	3.03 ± 1.71	5 ± 0	2.78 ± 1.72
		10	10 ± 0	8.48 ± 2.54	10 ± 0	4.23 ± 3.4	10 ± 0	3.51 ± 2.96
	2500	3	3 ± 0	2.89 ± 0.42	3 ± 0	2.26 ± 0.89	3 ± 0	2.1 ± 0.96
		5	5 ± 0	4.65 ± 0.98	5 ± 0	3.1 ± 1.72	5 ± 0	2.81 ± 1.73
		10	10 ± 0	8.35 ± 2.75	10 ± 0	4.35 ± 3.41	10 ± 0	3.51 ± 2.9
	5000	3	3 ± 0	2.9 ± 0.39	3 ± 0	2.25 ± 0.89	3 ± 0	2.09 ± 0.96
		5	5 ± 0	4.66 ± 0.94	5 ± 0	3.07 ± 1.71	5 ± 0	2.78 ± 1.73
		10	10 ± 0	8.37 ± 2.71	10 ± 0	4.29 ± 3.4	10.0 ± 0.04	3.48 ± 2.89
	7500	3	3 ± 0	2.91 ± 0.38	3 ± 0	2.24 ± 0.89	3 ± 0	2.07 ± 0.96
		5	5 ± 0	4.68 ± 0.92	5 ± 0	3.06 ± 1.72	5 ± 0	2.76 ± 1.73
		10	10 ± 0	8.44 ± 2.66	10.0 ± 0.01	4.31 ± 3.44	10.0 ± 0.01	3.42 ± 2.87
	10000	3	3 ± 0	2.9 ± 0.39	3 ± 0	2.24 ± 0.89	3 ± 0	2.08 ± 0.96
		5	5 ± 0	4.67 ± 0.93	5 ± 0	3.07 ± 1.71	5 ± 0	2.78 ± 1.73
		10	10 ± 0	8.43 ± 2.68	10 ± 0.03	4.3 ± 3.41	10.0 ± 0.01	3.46 ± 2.89
**p = 0.15**	1000	3	3 ± 0	1.76 ± 0.9	3 ± 0	2.58 ± 0.75	3 ± 0	2.22 ± 0.97
		5	5 ± 0	2.2 ± 1.6	5 ± 0	3.88 ± 1.59	5 ± 0	3.32 ± 1.88
		10	10 ± 0	2.93 ± 3.07	10 ± 0	6.48 ± 3.72	10 ± 0	4.46 ± 3.4
	2500	3	3 ± 0	1.77 ± 0.89	3 ± 0	2.58 ± 0.76	3 ± 0	2.22 ± 0.97
		5	5 ± 0	2.2 ± 1.58	5 ± 0	3.87 ± 1.59	5 ± 0	3.3 ± 1.87
		10	10 ± 0	2.88 ± 2.98	10 ± 0	6.39 ± 3.72	10 ± 0	4.43 ± 3.39
	5000	3	3 ± 0	1.75 ± 0.89	3 ± 0	2.58 ± 0.76	3 ± 0	2.2 ± 0.98
		5	5 ± 0	2.18 ± 1.58	5 ± 0	3.86 ± 1.59	5 ± 0	3.25 ± 1.89
		10	10 ± 0	2.88 ± 3.03	10 ± 0	6.35 ± 3.72	10 ± 0	4.37 ± 3.4
	7500	3	3 ± 0	1.78 ± 0.89	3 ± 0	2.57 ± 0.76	3 ± 0	2.19 ± 0.98
		5	5 ± 0	2.22 ± 1.59	5 ± 0	3.85 ± 1.6	5 ± 0	3.24 ± 1.89
		10	10 ± 0	2.93 ± 3.05	10 ± 0	6.34 ± 3.73	10 ± 0	4.36 ± 3.41
	10000	3	3 ± 0	1.77 ± 0.89	3 ± 0	2.58 ± 0.75	3 ± 0	2.19 ± 0.98
		5	5 ± 0	2.2 ± 1.58	5 ± 0	3.89 ± 1.58	5 ± 0	3.24 ± 1.89
		10	10 ± 0	2.9 ± 3.02	10 ± 0	6.42 ± 3.71	10 ± 0	4.36 ± 3.41
**p = 0.3**	1000	3	3 ± 0	1.17 ± 0.49	3 ± 0	2.62 ± 0.73	3.0 ± 0.07	2.23 ± 0.97
		5	5 ± 0	1.22 ± 0.72	5 ± 0	4.01 ± 1.54	4.98 ± 0.22	3.42 ± 1.94
		10	10 ± 0	1.31 ± 1.24	10 ± 0	6.78 ± 3.66	9.9 ± 0.79	4.62 ± 3.47
	2500	3	3 ± 0	1.18 ± 0.51	3 ± 0	2.62 ± 0.72	2.99 ± 0.14	2.23 ± 0.97
		5	5 ± 0	1.24 ± 0.76	5 ± 0	3.96 ± 1.54	4.97 ± 0.33	3.41 ± 1.93
		10	10 ± 0	1.32 ± 1.27	10 ± 0	6.67 ± 3.69	9.87 ± 0.94	4.61 ± 3.46
	5000	3	3 ± 0	1.18 ± 0.51	3 ± 0	2.62 ± 0.73	2.99 ± 0.12	2.2 ± 0.98
		5	5 ± 0	1.25 ± 0.82	5 ± 0	3.96 ± 1.54	4.97 ± 0.29	3.36 ± 1.94
		10	10 ± 0	1.35 ± 1.35	10 ± 0	6.62 ± 3.68	9.89 ± 0.86	4.55 ± 3.47
	7500	3	3 ± 0	1.18 ± 0.52	3 ± 0	2.62 ± 0.72	2.99 ± 0.13	2.19 ± 0.98
		5	5 ± 0	1.25 ± 0.8	5 ± 0	3.95 ± 1.54	4.97 ± 0.3	3.34 ± 1.94
		10	10 ± 0	1.35 ± 1.35	10 ± 0	6.61 ± 3.68	9.89 ± 0.87	4.52 ± 3.48
	10000	3	3 ± 0	1.18 ± 0.5	3 ± 0	2.63 ± 0.72	2.99 ± 0.14	2.2 ± 0.98
		5	5 ± 0	1.24 ± 0.78	5 ± 0	3.98 ± 1.53	4.96 ± 0.33	3.35 ± 1.94
		10	10 ± 0	1.33 ± 1.3	10 ± 0	6.67 ± 3.67	9.87 ± 0.94	4.53 ± 3.47

Supporting Table S2. Distribution learning of physico-chemical properties. We report the Kolmogorov-Smirnov (KS) distance between the de novo designs (4000 SMILES strings) and the training set molecules, computed for four rest descriptors (number of aliphatic or aromatic rings, rotatable bonds, and TPSA= topological surface). For each training set size (1000, 2500, 5000, 7500, and 10000 molecules), the KS distance is reported for each augmentation strategy, and each descriptor. For each descriptor and training set size, the best and second-best KS distances are highlighted in bold and underlined, respectively. The number of times a given augmentation strategy provides the best or second-best performance for a given descriptor across training set sizes is also reported. The KS distances between the training and the test set molecules, and for the designs obtained with no augmentation are reported as a reference (n.a. = not available).

**Property**	**Method**	**Training Set Size**					**Times Top-2**
		**1000**	**2500**	**5000**	**7500**	**10000**	
**No. Aliphatic Rings**	Enumeration	7 ± 3	8.3 ± 0.5	**3 ± 2**	7 ± 1	**3.3 ± 0.2**	2
	Token Deletion (Random)	8 ± 2	15 ± 8	11 ± 4	8.7 ± 0.9	10 ± 6	0
	Token Deletion (Validity)	8 ± 3	12 ± 3	14.6 ± 0.9	9 ± 1	15 ± 2	0
	Token Deletion (Protected)	16 ± 3	17 ± 3	16 ± 3	7 ± 3	12 ± 2	0
	Atom Masking (Random)	7 ± 2	6 ± 2	5 ± 1	**5 ± 1**	4 ± 1	3
	Atom Masking (Funct. Group)	6 ± 3	**4.8 ± 0.7**	4 ± 1	5 ± 4	8 ± 2	4
	Bioisosteric Substitution	11.9 ± 0.3	14 ± 2	18 ± 2	10 ± 2	8.5 ± 0.6	0
	Self-Training	8.8 ± 0.9	8 ± 2	4.4 ± 0.8	8 ± 1	4 ± 1	1
	No augmentation	**5 ± 2**	10.1 ± 1.0	9.8 ± 0.2	8 ± 1	6 ± 2	1
	Train - Test	2	3	2	1	2	n.a.
**No. Aromatic Rings**	Enumeration	17 ± 2	16.3 ± 0.8	8 ± 1	**5.4 ± 0.9**	7 ± 1	3
	Token Deletion (Random)	37 ± 4	25 ± 2	31 ± 3	28 ± 5	26 ± 3	0
	Token Deletion (Validity)	31 ± 4	25 ± 2	24 ± 1	21.9 ± 0.4	26 ± 1	0
	Token Deletion (Protected)	46 ± 3	31.5 ± 0.7	27 ± 3	30 ± 2	27 ± 4	0
	Atom Masking (Random)	21 ± 2	23 ± 1	12 ± 2	11.6 ± 0.6	6 ± 1	1
	Atom Masking (Funct. Group)	21 ± 4	**14 ± 5**	8 ± 1	10 ± 2	8 ± 2	3
	Bioisosteric Substitution	**11 ± 2**	17 ± 2	**5 ± 2**	10 ± 2	**2.4 ± 0.4**	**4**
	Self-Training	37 ± 2	22.3 ± 0.4	23 ± 2	16 ± 2	20 ± 1	0
	No augmentation	39 ± 4	16 ± 2	10 ± 2	17 ± 1	13.3 ± 0.5	1
	Train - Test	4	4	4	4	3	n.a.
**Rotatable Bonds**	Enumeration	**5 ± 1**	5 ± 3	**3.0 ± 0.5**	**3.6 ± 0.7**	**2.2 ± 0.7**	**5**
	Token Deletion (Random)	22 ± 8	14 ± 1	15 ± 2	15 ± 4	19 ± 3	0
	Token Deletion (Validity)	6.1 ± 0.5	**5 ± 2**	8 ± 1	6.0 ± 0.4	9 ± 1	2
	Token Deletion (Protected)	21 ± 11	11 ± 2	7 ± 4	10 ± 1	10 ± 3	0
	Atom Masking (Random)	10 ± 2	**5 ± 2**	5 ± 3	5 ± 2	6 ± 1	2
	Atom Masking (Funct. Group)	14 ± 1	11 ± 3	4 ± 2	8 ± 3	6 ± 1	1
	Bioisosteric Substitution	20 ± 1	18 ± 2	23 ± 3	22.2 ± 0.9	22.5 ± 0.4	0
	Self-Training	23 ± 1	11 ± 1	21 ± 3	16 ± 2	18.8 ± 0.8	0
	No Augmentation	19 ± 6	8.7 ± 0.9	4 ± 3	5 ± 2	4 ± 1	2
	Train - Test	4	3	2	2	2	n.a.
**TPSA**	Enumeration	**6 ± 2**	10.4 ± 0.4	**3.3 ± 0.4**	**5 ± 1**	**3.1 ± 0.1**	**4**
	Token Deletion (Random)	27 ± 8	27 ± 5	21 ± 2	21 ± 9	25 ± 4	0
	Token Deletion (Validity)	17 ± 2	17 ± 4	13.8 ± 0.6	12.9 ± 0.2	18 ± 2	0
	Token Deletion (Protected)	33 ± 14	17 ± 2	16 ± 4	18 ± 1	18 ± 3	0
	Atom Masking (Random)	18 ± 5	19 ± 2	11 ± 2	10 ± 3	11 ± 3	0
	Atom Masking (Funct. Group)	8 ± 3	**8 ± 2**	8 ± 4	**5 ± 2**	7 ± 2	2
	Bioisosteric Substitution	7 ± 2	11.0 ± 0.9	4 ± 2	6 ± 1	6 ± 1	**4**
	Self-Training	43 ± 2	9 ± 2	6 ± 2	11.7 ± 0.8	8 ± 2	1
	No Augmentation	34 ± 10	16 ± 2	13.8 ± 0.8	10 ± 4	13 ± 1	0
	Train -Test	4	3	3	3	3	n.a.

Supporting Table S3. Augmentation folds used for bioisosteric substitution for each fine-tuning set with different dataset sizes and similarity (high/low) at different targets. If the augmentation fold is 10, then for 100 starting molecules the augmented set would be 1000 molecules.

Fine-tuning set		PPARδ		PIM1		JAK1	
**Similarity**	**Size**	**Bioisosteric Substitution**	**Masking (Funct. Group)**	**Bioisosteric Substitution**	**Masking (Funct. Group)**	**Bioisosteric Substitution**	**Masking (Funct. Group)**
**High**	10	10	10 ± 0	2.5 ± 2.07	6.38 ± 3.74	8.12 ± 2.59	10 ± 0
	100	9.94 ± 0.56	10 ± 0	1.9 ± 1.68	6.85 ± 3.27	5.96 ± 2.08	10 ± 0
**Low**	10	7.12 ± 3.4	10 ± 0	4.62 ± 2.83	8.88 ± 3.18	5.88 ± 3.9	9.25 ± 2.12
	100	7.36 ± 3.15	10 ± 0	3.84 ± 3.3	8.54 ± 2.9	4.92 ± 3.75	8.7 ± 2.79

Supporting Table S4. Validity, uniqueness, and novelty of the sampled molecules after training with various fine-tuning set (similarity: high, low, size: 10, 100) for different targets (PPARδ, PIM1, JAK2). Results after training with no augmentation are reported as a baseline.

Similarity	Target	Method	10 fine-tuning molecules			100 fine-tuning molecules		
			Validity (%)	Uniqueness (%)	Novelty (%)	Validity (%)	Uniqueness (%)	Novelty (%)
**High**	**PPARδ**	Enumeration	89.5 ± 0.7	99.5 ± 0.4	99.6 ± 0.1	87.5 ± 0.6	78 ± 1	90.2 ± 0.4
		Deletion (Random)	83.5 ± 0.6	92.9 ± 0.8	99.0 ± 0.1	40.1 ± 0.6	74 ± 1	80.5 ± 0.9
		Deletion (Validity)	93.1 ± 0.4	84 ± 2	99 ± 0	85 ± 2	78 ± 2	90.6 ± 1.0
		Deletion (Protected)	74.5 ± 0.5	87.6 ± 0.4	99 ± 0	38 ± 2	77 ± 4	79.5 ± 0.9
		Masking (Random)	94.1 ± 0.5	54 ± 2	98 ± 0	96.9 ± 0.6	30.7 ± 0.5	73.2 ± 0.5
		Masking (Funct. Group)	94.2 ± 0.8	79 ± 2	99 ± 0	98.4 ± 0.3	35 ± 1	77 ± 1
		Bioisosteric Substitution	93.9 ± 0.7	92 ± 2	99.1 ± 0.1	97.1 ± 0.3	89 ± 1	94.7 ± 0.7
		Self-Training	95.2 ± 0.8	99.7 ± 0.3	100 ± 0.1	95.8 ± 0.6	95.6 ± 0.5	97.3 ± 0.2
		No Augmentation	92.6 ± 0.5	100 ± 0	100 ± 0	94.5 ± 0.6	83 ± 2	91.7 ± 0.3
	**PIM1**	Enumeration	84 ± 1	99.7 ± 0.2	99.5 ± 0.3	69.6 ± 0.9	81 ± 2	89.6 ± 0.7
		Deletion (Random)	83.6 ± 0.1	98.1 ± 0.3	99.2 ± 0.1	36.9 ± 0.3	78.9 ± 0.9	79.7 ± 0.9
		Deletion (Validity)	88 ± 1	99.2 ± 0.1	99.6 ± 0.2	80.0 ± 0.6	67.8 ± 0.8	87.2 ± 0.7
		Deletion (Protected)	79 ± 2	99.0 ± 0.3	99.5 ± 0.2	33.9 ± 0.8	78 ± 2	79 ± 2
		Masking (Random)	88.6 ± 1.0	85 ± 2	98.9 ± 0.0	95.6 ± 0.5	30.8 ± 0.4	72.9 ± 0.2
		Masking (Funct. Group)	94.9 ± 0.6	80 ± 1	99.1 ± 0.0	95.7 ± 0.5	46 ± 1	83 ± 1
		Bioisosteric Substitution	91.6 ± 0.6	99.5 ± 0.5	99.6 ± 0.2	94.0 ± 0.2	77 ± 2	91.5 ± 0.3
		Self-Training	93.4 ± 0.7	100.0 ± 0.1	100 ± 0	93.9 ± 0.5	95.8 ± 0.4	97.8 ± 0.5
		No Augmentation	92.8 ± 0.3	100 ± 0	99.9 ± 0.2	93.3 ± 0.4	80.4 ± 0.8	90.7 ± 0.4
	**JAK2**	Enumeration	77 ± 2	99.9 ± 0.1	99.7 ± 0.1	56 ± 1	87 ± 2	88.5 ± 0.7
		Deletion (Random)	81.4 ± 0.8	91.0 ± 0.8	99.0 ± 0.1	40 ± 3	88.5 ± 0.3	85.2 ± 0.6
		Deletion (Validity)	87.7 ± 0.4	82.4 ± 1.0	98.9 ± 0.0	86.0 ± 0.4	82 ± 1	91.6 ± 0.4
		Deletion (Protected)	71.9 ± 0.3	92.0 ± 0.5	98.8 ± 0.1	45 ± 1	82 ± 2	83.7 ± 0.2
		Masking (Random)	95.5 ± 0.5	37 ± 1	97.7 ± 0.1	96.4 ± 0.4	38 ± 2	78.3 ± 1.0
		Masking (Funct. Group)	95.7 ± 0.8	40 ± 1	97.9 ± 0.1	96.2 ± 0.7	45.5 ± 0.3	82.1 ± 0.5
		Bioisosteric Substitution	93 ± 1	81.1 ± 0.8	98.9 ± 0.0	95.7 ± 0.3	82.2 ± 0.9	93.7 ± 0.2
		Self-Training	93.1 ± 0.3	99.1 ± 0.4	99.9 ± 0.2	95.5 ± 0.4	97.1 ± 0.2	97.7 ± 0.2
		No Augmentation	92.5 ± 0.7	99.9 ± 0.1	99.6 ± 0.1	94.5 ± 0.9	79.8 ± 1.0	91.4 ± 0.6
**Low**	**PPARδ**	Enumeration	85.5 ± 0.8	99.8 ± 0.2	99.9 ± 0.1	71 ± 2	99.9 ± 0.1	99.9 ± 0.1
		Deletion (Random)	88 ± 1	98.1 ± 0.3	99.2 ± 0.0	61.2 ± 0.8	97.5 ± 1.0	95.2 ± 0.4
		Deletion (Validity)	91 ± 1	99.6 ± 0.3	99.7 ± 0.0	82 ± 2	99.2 ± 0.5	97.7 ± 0.4
		Deletion (Protected)	84 ± 2	99.9 ± 0.1	99.8 ± 0.1	59 ± 2	99.7 ± 0.3	98.3 ± 0.2
		Masking (Random)	91.8 ± 0.2	99.9 ± 0.1	99.7 ± 0.1	87.2 ± 0.7	98.2 ± 0.1	97.1 ± 0.1
		Masking (Funct. Group)	94.5 ± 1.1	68.3 ± 0.8	98.8 ± 0.0	90.8 ± 0.2	96.0 ± 0.3	96.1 ± 0.6
		Bioisosteric Substitution	92.5 ± 1.0	99.9 ± 0.1	99.7 ± 0.1	92.2 ± 0.5	100.0 ± 0.1	99.7 ± 0.3
		Self-Training	94.4 ± 1.0	100 ± 0	100 ± 0	95.8 ± 0.5	99.0 ± 0.3	99.9 ± 0.1
		No Augmentation	92.2 ± 0.6	100 ± 0	100 ± 0	93.5 ± 0.6	100.0 ± 0.1	99.5 ± 0.2
	**PIM1**	Enumeration	83.2 ± 0.5	99.9 ± 0.1	99.8 ± 0.1	58.6 ± 1.0	99.1 ± 0.1	98.0 ± 0.3
		Deletion (Random)	86.5 ± 0.6	98.1 ± 0.3	99.3 ± 0.1	66 ± 1	98.2 ± 0.4	96.6 ± 0.3
		Deltion (Validity)	88 ± 1	99.9 ± 0.1	99.9 ± 0.1	73.0 ± 0.6	99.5 ± 0.3	98.8 ± 0.1
		Deletion (Protected)	86.0 ± 0.6	100 ± 0	99.9 ± 0.1	51 ± 1	99.7 ± 0.1	98.3 ± 0.9
		Masking (Random)	89 ± 1	99.7 ± 0.1	99.6 ± 0.1	81 ± 2	98.0 ± 0.2	96.5 ± 0.3
		Masking (Funct. Group)	90.5 ± 0.4	99.5 ± 0.3	99.6 ± 0.1	90 ± 1	92.9 ± 0.9	94.9 ± 0.3
		Bioisosteric Substitution	92 ± 1	100.0 ± 0.1	99.9 ± 0.1	91.7 ± 0.9	100 ± 0	99.8 ± 0.2
		Self-Training	94 ± 2	100.0 ± 0.1	100 ± 0	94.0 ± 0.6	100.0 ± 0.1	100 ± 0
		No Augmentation	91.8 ± 0.7	100.0 ± 0.1	100 ± 0	91.2 ± 0.8	100.0 ± 0.1	99.8 ± 0.1
	**JAK2**	Enumeration	88 ± 2	99.9 ± 0.1	99.9 ± 0.0	65 ± 2	99.5 ± 0.5	98.4 ± 0.2
		Deletion (Random)	87 ± 1	98.4 ± 0.9	99.3 ± 0.1	65 ± 2	98.9 ± 0.1	96.7 ± 1.0
		Deltion (Validity)	91.3 ± 0.8	99.9 ± 0.1	99.9 ± 0.1	76 ± 1	99.5 ± 0.3	98.8 ± 0.4
		Deletion (Protected)	79.1 ± 0.8	98.9 ± 0.5	99.3 ± 0.1	66.6 ± 0.7	100 ± 0	99.9 ± 0.2
		Masking (Random)	88.1 ± 0.8	97.7 ± 0.5	99.3 ± 0.1	85.7 ± 1.0	96.5 ± 0.5	95.5 ± 0.7
		Masking (Funct. Group)	91.3 ± 0.9	98.7 ± 0.6	99.4 ± 0.1	88 ± 2	98.4 ± 0.5	97.1 ± 0.3
		Bioisosteric Substitution	93 ± 2	100.0 ± 0.1	100 ± 0	90.9 ± 0.8	100.0 ± 0.1	99.9 ± 0.2
		Self-Training	94 ± 1	99.9 ± 0.2	100 ± 0	94.3 ± 0.4	100.0 ± 0.1	100 ± 0
		No Augmentation	92 ± 1	100.0 ± 0.1	99.9 ± 0.1	92 ± 2	100.0 ± 0.1	99.9 ± 0.1

Supporting Table S5. The Kolmogorov-Smirnov (KS) distance between the de novo designs (1000 SMILES strings sampled with 4 repetitions) and the fine-tuning set molecules of the target PPARδ, computed for selected

descriptors. For each training set size (10, 100 molecules) and similarities (high, low), the KS distance is reported for each augmentation strategy, and each descriptor. For each descriptor and training set size, the best and second-best KS distances are highlighted in bold and underlined, respectively.

Property	Method	High similarity		High dissimilarity	
		10	100	10	100
**No. Aliphatic Rings**	Enumeration	10.7 ± 0.7	6.2 ± 0.8	29.8 ± 0.9	10.4 ± 0.5
	Token Deletion (Random)	12.2 ± 0.6	9 ± 2	35 ± 2	10 ± 2
	Token Deletion (Validity)	14.2 ± 0.4	8.0 ± 0.9	49 ± 2	12 ± 2
	Token Deletion (Protected)	23.12 ± 0.4	10 ± 2	54 ± 1	18.6 ± 1.0
	Atom Masking (Random)	13.97 ± 0.6	4 ± 1	49.3 ± 0.9	12 ± 2
	Atom Masking (Funct. Group)	17 ± 2	1.8 ± 0.4	37 ± 3	10 ± 2
	Bioisosteric Substitution	13.1 ± 0.9	6.7 ± 0.6	49 ± 3	11.0 ± 0.8
	Self-Training	36 ± 3	5 ± 1	54 ± 2	13 ± 1
	No Augmentation	42 ± 0.9	5.0 ± 0.6	60 ± 3	18 ± 2
**No. Aromatic Rings**	Enumeration	40 ± 2	14 ± 1	49.4 ± 0.2	20.1 ± 0.9
	Token Deletion (Random)	16 ± 1	20 ± 2	38 ± 2	31 ± 1
	Token Deletion (Validity)	13 ± 3	10 ± 2	38 ± 2	23 ± 2
	Token Deletion (Protected)	18 ± 2	21 ± 3	41 ± 4	29.4 ± 0.3
	Atom Masking (Random)	14 ± 1	12.6 ± 0.8	39 ± 3	27 ± 2
	Atom Masking (Funct. Group)	19 ± 2	9 ± 1	35 ± 1	22.6 ± 0.5
	Bioisosteric Substitution	12 ± 2	9.2 ± 0.9	39.8 ± 1.0	13 ± 1
	Self-Training	37 ± 1	40.0 ± 0.9	48 ± 1	40.5 ± 0.6
	No Augmentation	25 ± 2	17 ± 2	40 ± 3	25 ± 3
**HBA**	Enumeration	25 ± 1	16.2 ± 0.5	31 ± 1	11 ± 3
	Token Deletion (Random)	28 ± 2	21 ± 4	39 ± 1	16 ± 2
	Token Deletion (Validity)	27 ± 2	18 ± 2	36 ± 1	14 ± 3
	Token Deletion (Protected)	24 ± 2	21 ± 4	37 ± 1	15 ± 2
	Atom Masking (Random)	28 ± 3	17 ± 1	36 ± 2	13 ± 3
	Atom Masking (Funct. Group)	26 ± 1	13 ± 5	44 ± 1	12 ± 1
	Bioisosteric Substitution	22 ± 0.8	7.9 ± 0.8	34.9 ± 0.5	10 ± 1
	Self-Training	32 ± 2	20 ± 1	39 ± 2	17.3 ± 0.6
	No Augmentation	22 ± 1	15 ± 2	29.7 ± 0.3	9 ± 1
**HBD**	Enumeration	27.8 ± 1.0	20.0 ± 0.4	39 ± 1	15 ± 1
	Token Deletion (Random)	27 ± 2	20 ± 1	28 ± 1	17 ± 1
	Token Deletion (Validity)	24 ± 1	14 ± 3	34.7 ± 1.0	15.6 ± 0.9
	Token Deletion (Protected)	23 ± 2	18 ± 1	34 ± 2	15.5 ± 1.0
	Atom Masking (Random)	25 ± 2	21 ± 4	35 ± 2	15.1 ± 0.2
	Atom Masking (Funct. Group)	23.9 ± 0.7	21 ± 1	30 ± 1	18 ± 1
	Bioisosteric Substitution	32 ± 2	28.4 ± 0.3	41 ± 1	16 ± 2
	Self-Training	34 ± 2	27.6 ± 0.3	44 ± 1	19.4 ± 0.6
	No Augmentation	29.6 ± 0.7	25.2 ± 0.7	39.9 ± 0.8	19 ± 1
**Rot. Bonds**	Enumeration	74 ± 1	27 ± 2	55 ± 2	21 ± 1
	Token Deletion (Random)	62 ± 2	41 ± 3	51 ± 1	29 ± 2
	Token Deletion (Validity)	62 ± 1	33 ± 3	51 ± 1	33 ± 2
	Token Deletion (Protected)	66 ± 2	37 ± 2	56 ± 2	38 ± 2
	Atom Masking (Random)	50 ± 3	27 ± 2	51 ± 2	29 ± 2
	Atom Masking (Funct. Group)	58 ± 2	28 ± 2	51 ± 2	27 ± 2
	Bioisosteric Substitution	44.8 ± 0.4	16.0 ± 0.5	42 ± 2	24 ± 1
	Self-Training	68 ± 3	36.4 ± 0.6	49 ± 2	24.7 ± 0.2
	No Augmentation	75 ± 1	50.1 ± 0.8	55 ± 3	38.6 ± 0.8
**MW**	Enumeration	71 ± 1	24 ± 2	52.3 ± 0.4	33 ± 1
	Token Deletion (Random)	55 ± 3	33 ± 2	48.2 ± 0.5	43 ± 2
	Token Deletion (Validity)	54 ± 1	22 ± 3	47 ± 1	40 ± 2
	Token Deletion (Protected)	54 ± 1	33 ± 2	49 ± 2	47.6 ± 0.6
	Atom Masking (Random)	45 ± 2	24 ± 2	46.9 ± 0.8	39 ± 2
	Atom Masking (Funct. Group)	52 ± 2	24 ± 1	48 ± 2	36 ± 1
	Bioisosteric Substitution	41 ± 2	14 ± 1	41 ± 3	27 ± 1
	Self-Training	66 ± 2	40.1 ± 0.3	47 ± 1	37 ± 2
	No Augmentation	63.7 ± 0.5	39 ± 1	47.8 ± 0.8	43.2 ± 0.6
**logP**	Enumeration	67.1 ± 0.5	23 ± 2	65.1 ± 0.5	25 ± 1
	Token Deletion (Random)	52 ± 3	30 ± 3	53 ± 2	35 ± 2
	Token Deletion (Validity)	49.2 ± 0.9	17 ± 2	57 ± 2	32 ± 1
	Token Deletion (Protected)	52.5 ± 0.7	30 ± 4	61 ± 2	41 ± 1
	Atom Masking (Random)	42.8 ± 0.2	24 ± 5	59.2 ± 0.5	33.6 ± 0.9
	Atom Masking (Funct. Group)	54 ± 3	21 ± 2	51.1 ± 1.0	30.2 ± 0.8
	Bioisosteric Substitution	44 ± 2	19 ± 2	56 ± 2	26 ± 2
	Self-Training	67 ± 2	41.4 ± 0.8	63 ± 2	37 ± 1

	No Augmentation	69.1 ± 0.3	42 ± 2	67 ± 1	43.2 ± 0.3
**TPSA**	Enumeration	34 ± 2	30 ± 1	25.5 ± 0.4	26 ± 3
	Token Deletion (Random)	38 ± 2	33 ± 2	35 ± 2	31 ± 2
	Token Deletion (Validity)	34 ± 2	23.9 ± 0.5	30.2 ± 0.8	28 ± 4
	Token Deletion (Protected)	33.6 ± 0.9	23 ± 1	30 ± 1	29 ± 2
	Atom Masking (Random)	36 ± 2	27 ± 2	28 ± 2	26 ± 2
	Atom Masking (Funct. Group)	30.0 ± 0.5	25 ± 3	36 ± 2	26 ± 1
	Bioisosteric Substitution	31 ± 1	23 ± 3	29.1 ± 1.0	21 ± 1
	Self-Training	33 ± 1	31.9 ± 0.5	28 ± 2	22.5 ± 0.2
	No Augmentation	35 ± 1	32 ± 2	24 ± 1	26 ± 2

Supporting Table S6. We report the Kolmogorov-Smirnov (KS) distance between the de novo designs (1000 SMILES strings sampled with 4 repetitions) and the fine-tuning set molecules of the target PIM1, computed for selected descriptors. For each training set size (10, 100 molecules) and similarities (high, low), the KS distance is reported for each augmentation strategy, and each descriptor. For each descriptor and training set size, the best and second-best KS distances are highlighted in bold and underlined, respectively.

Property	Method	Highly similar		High Dissimilar	
		10	100	10	100
**No. Aliphatic Rings**	Enumeration	11.4 ± 0.7	9.9 ± 0.6	37 ± 2	13.7 ± 1.0
	Token Deletion (Random)	12 ± 1	15.8 ± 0.3	39 ± 2	10.6 ± 0.7
	Token Deletion (Validity)	15.8 ± 0.9	24 ± 1	52.9 ± 0.2	23 ± 1
	Token Deletion (Protected)	19.4 ± 0.3	27 ± 2	58.3 ± 0.8	21 ± 2
	Atom Masking (Random)	11.5 ± 0.4	12 ± 3	54 ± 4	17.7 ± 0.6
	Atom Masking (Funct. Group)	11.1 ± 1.0	20 ± 3	49.3 ± 0.3	15 ± 2
	Bioisoster Substitution	15.7 ± 0.8	28 ± 2	54 ± 1	13 ± 2
	Self-Training	16.9 ± 0.3	11.4 ± 0.5	56 ± 2	15.7 ± 0.5
	No Augmentation	18.8 ± 0.9	12 ± 1	61 ± 1	23 ± 2
**No. Aromatic Rings**	Enumeration	68 ± 1	33 ± 3	43.7 ± 0.7	18 ± 3
	Token Deletion (Random)	62.2 ± 0.6	39.8 ± 0.9	31 ± 2	21 ± 2
	Token Deletion (Validity)	69.1 ± 0.7	36 ± 2	35.7 ± 1.0	20 ± 2
	Token Deletion (Protected)	66.2 ± 0.3	48 ± 2	40 ± 1	25.2 ± 0.5
	Atom Masking (Random)	57.6 ± 0.2	18 ± 1	37 ± 3	17.4 ± 0.6
	Atom Masking (Funct. Group)	63 ± 3	29 ± 3	35.1 ± 0.9	17 ± 2
	Bioisoster Substitution	73 ± 1	38 ± 2	39.5 ± 0.9	16 ± 3
	Self-Training	86.3 ± 0.9	57 ± 1	50.0 ± 0.4	28 ± 2
	No Augmentation	79.5 ± 0.7	54 ± 2	45.1 ± 0.2	22 ± 1
**HBA**	Enumeration	15.6 ± 0.8	16.8 ± 0.7	19.0 ± 0.7	11 ± 1
	Token Deletion (Random)	18 ± 2	22 ± 3	19.9 ± 0.8	11 ± 1
	Token Deletion (Validity)	15 ± 1	17 ± 3	20 ± 1	7.7 ± 0.3
	Token Deletion (Protected)	17 ± 1	25 ± 2	21.5 ± 0.4	8 ± 1
	Atom Masking (Random)	20 ± 1	22 ± 5	20 ± 2	7.6 ± 0.6
	Atom Masking (Funct. Group)	20 ± 2	29 ± 2	21 ± 1	5.0 ± 0.8
	Bioisoster Substitution	15 ± 4	29.2 ± 0.1	21.9 ± 0.8	13 ± 2
	Self-Training	19.0 ± 0.7	39 ± 1	21 ± 1	10 ± 2
	No Augmentation	13.5 ± 0.9	33.3 ± 0.5	22.0 ± 0.1	13.7 ± 0.4
**HBD**	Enumeration	15.6 ± 0.7	16 ± 3	16.2 ± 0.9	13.7 ± 0.4
	Token Deletion (Random)	17 ± 1	14 ± 4	15.1 ± 0.5	13.7 ± 0.5
	Token Deletion (Validity)	19.8 ± 0.9	20 ± 2	19 ± 2	14.6 ± 0.7
	Token Deletion (Protected)	19 ± 2	20 ± 3	21 ± 3	14.0 ± 0.4
	Atom Masking (Random)	16 ± 2	22 ± 2	18.3 ± 0.8	13.1 ± 0.2
	Atom Masking (Funct. Group)	14 ± 1	17 ± 5	19 ± 1	13.8 ± 0.7
	Bioisoster Substitution	18 ± 1	16 ± 2	19 ± 0.9	14.0 ± 0.7
	Self-Training	18.2 ± 0.6	27.6 ± 0.5	18 ± 2	14.6 ± 0.4
	No Augmentation	20 ± 2	27 ± 2	22 ± 1	16.5 ± 0.4
**Rot. Bonds**	Enumeration	14 ± 1	18 ± 1	14 ± 1	6.1 ± 0.9
	Token Deletion (Random)	14.9 ± 0.4	19 ± 5	16 ± 1	4.4 ± 0.5
	Token Deletion (Validity)	17 ± 2	10.7 ± 0.7	20.4 ± 0.5	6 ± 3
	Token Deletion (Protected)	16 ± 2	18 ± 1	22 ± 1	6 ± 2
	Atom Masking (Random)	18.5 ± 0.9	11 ± 3	22 ± 4	15 ± 3
	Atom Masking (Funct. Group)	19 ± 1	16 ± 2	24 ± 2	15 ± 1
	Bioisoster Substitution	29 ± 2	31 ± 2	27 ± 1	32 ± 1
	Self-Training	38 ± 2	30 ± 1	34 ± 2	35 ± 2
	No Augmentation	28 ± 1	18 ± 2	28 ± 2	23 ± 2
**MW**	Enumeration	38 ± 1	26.6 ± 0.4	28 ± 1	11 ± 3
	Token Deletion (Random)	37.0 ± 0.5	37 ± 2	30 ± 4	13 ± 1
	Token Deletion (Validity)	40 ± 2	31 ± 2	37 ± 0.7	7 ± 2
	Token Deletion (Protected)	43 ± 1	30 ± 3	37 ± 3	10 ± 3
	Atom Masking (Random)	44 ± 2	22 ± 3	40 ± 3	13 ± 0.8
	Atom Masking (Funct. Group)	35 ± 2	26 ± 3	42 ± 2	14 ± 2
	Bioisoster Substitution	45 ± 2	25 ± 1	41.9 ± 0.4	22 ± 2
	Self-Training	48.8 ± 0.9	24 ± 1	41 ± 2	21.0 ± 1.0
	No Augmentation	47.3 ± 0.2	34.4 ± 0.6	41.3 ± 0.4	17.4 ± 0.8
**logP**	Enumeration	31 ± 2	13.6 ± 0.8	22.2 ± 0.9	11 ± 1
	Token Deletion (Random)	28 ± 1	15 ± 2	34.4 ± 0.9	9 ± 1
	Token Deletion (Validity)	27 ± 1	12.9 ± 0.6	31.2 ± 0.9	9 ± 2
	Token Deletion (Protected)	29 ± 1	14 ± 3	29 ± 1	11 ± 2
	Atom Masking (Random)	34 ± 2	18 ± 2	33 ± 2	12 ± 1
	Atom Masking (Funct. Group)	27.6 ± 0.4	20 ± 2	34.2 ± 0.6	14 ± 2
	Bioisoster Substitution	27.6 ± 0.7	19 ± 2	31.7 ± 0.7	19 ± 2
	Self-Training	30.3 ± 0.3	31 ± 2	30 ± 1	19 ± 2

	No Augmentation	28.0 ± 0.7	19 ± 1	27 ± 3	11 ± 2
**TPSA**	Enumeration	24.0 ± 0.5	31 ± 1	27 ± 2	22.18 ± 0.6
	Token Deletion (Random)	28 ± 2	32 ± 2	30.7 ± 0.5	21.5 ± 0.4
	Token Deletion (Validity)	30 ± 1	32 ± 3	29 ± 2	18 ± 2
	Token Deletion (Protected)	28.7 ± 0.4	40 ± 2	30 ± 2	16 ± 3
	Atom Masking (Random)	29 ± 1	29 ± 2	28 ± 2	15 ± 3
	Atom Masking (Funct. Group)	23 ± 2	26 ± 2	25 ± 2	13 ± 1
	Bioisoster Substitution	25 ± 2	31 ± 1	26 ± 2	10 ± 1
	Self-Training	29 ± 2	44 ± 1	30 ± 1	15 ± 2
	No Augmentation	27 ± 2	42.0 ± 1.0	25 ± 1	11 ± 1.0

Supporting Table S7. We report the Kolmogorov-Smirnov (KS) distance between the de novo designs (1000 SMILES strings sampled with 4 repetitions) and the fine-tuning set molecules of the target JAK2, computed for selected descriptors. For each training set size (10, 100 molecules) and similarities (high, low), the KS distance is reported for each augmentation strategy, and each descriptor. For each descriptor and training set size, the best and second-best KS distances are highlighted in bold and underlined, respectively.

Property	Method	Highly Similar		Highly Dissimilar	
		10	100	10	100
No. Aliphatic Rings	Enumeration	43.5 ± 0.7	11 ± 1	25.2 ± 1.0	7 ± 3
	Token Deletion (Random)	27 ± 2	9 ± 2	36 ± 2	6.3 ± 1.0
	Token Deletion (Validity)	14 ± 2	6.5 ± 0.5	40.8 ± 1.0	13 ± 2
	Token Deletion (Protected)	17 ± 3	10 ± 5	40 ± 1	18 ± 2
	Atom Masking (Random)	18 ± 3	11 ± 3	40.2 ± 0.8	12 ± 1
	Atom Masking (Funct. Group)	22 ± 5	13.5 ± 0.7	39 ± 1	14 ± 2
	Bioisosteric Substitution	27 ± 3	8.7 ± 0.3	45 ± 2	8.8 ± 0.6
	Self-Training	37 ± 2	14 ± 2	41 ± 3	9 ± 2
	No Augmentation	36.8 ± 0.6	10.2 ± 0.4	43 ± 3	16 ± 1
No. Aromatic Rings	Enumeration	54.4 ± 0.8	47 ± 4	31.2 ± 1.0	23 ± 2
	Token Deletion (Random)	52 ± 2	57.1 ± 0.8	16 ± 3	23 ± 2
	Token Deletion (Validity)	50 ± 2	33 ± 2	23.8 ± 0.5	24 ± 1
	Token Deletion (Protected)	50 ± 2	46 ± 4	23 ± 1	28 ± 2
	Atom Masking (Random)	52 ± 2	37.2 ± 0.6	20 ± 2	23 ± 1
	Atom Masking (Funct. Group)	48.4 ± 0.9	45 ± 1	18 ± 2	23 ± 1
	Bioisosteric Substitution	48.5 ± 0.9	38.8 ± 0.4	26 ± 3	23 ± 1
	Self-Training	69.5 ± 0.6	70.9 ± 0.3	36 ± 2	38 ± 1
	No Augmentation	58 ± 1	67.5 ± 0.9	26 ± 1	29 ± 2
HBA	Enumeration	56 ± 2	42 ± 3	12.7 ± 0.3	12 ± 2
	Token Deletion (Random)	60 ± 1	50.7 ± 1.0	19 ± 2	10.4 ± 0.4
	Token Deletion (Validity)	55 ± 3	24 ± 1	21.1 ± 0.5	10.4 ± 0.6
	Token Deletion (Protected)	60 ± 1	36 ± 2	22.8 ± 0.5	9 ± 2
	Atom Masking (Random)	56 ± 2	33 ± 1	19 ± 2	9 ± 2
	Atom Masking (Funct. Group)	54.8 ± 0.8	40 ± 3	21 ± 1	5.3 ± 0.6
	Bioisosteric Substitution	55 ± 2	29.4 ± 0.4	23 ± 2	10.9 ± 0.3
	Self-Training	66 ± 1	58 ± 2	14.4 ± 0.3	12 ± 3
	No Augmentation	65.7 ± 0.7	58 ± 2	21 ± 2	6 ± 1
HBD	Enumeration	18 ± 3	13 ± 2	30 ± 2	15.1 ± 0.6
	Token Deletion (Random)	13.8 ± 0.8	13 ± 3	32.6 ± 0.8	19.6 ± 1.0
	Token Deletion (Validity)	14 ± 1	9.8 ± 0.9	38 ± 2	20 ± 2
	Token Deletion (Protected)	14.3 ± 0.5	14 ± 2	32 ± 1	23 ± 3
	Atom Masking (Random)	14 ± 4	11 ± 2	35 ± 1	18 ± 2
	Atom Masking (Funct. Group)	11 ± 1	15 ± 3	32.4 ± 0.9	20 ± 1
	Bioisosteric Substitution	19 ± 1	24 ± 3	39.7 ± 0.4	21.1 ± 0.3
	Self-Training	16.3 ± 0.4	15.2 ± 0.2	35.1 ± 0.1	21 ± 2
	No Augmentation	18.7 ± 0.9	18.5 ± 0.7	42 ± 3	27.2 ± 0.3
Rot. Bonds	Enumeration	45 ± 1	26 ± 3	23.7 ± 1.0	11.3 ± 0.8
	Token Deletion (Random)	48.6 ± 0.6	37 ± 2	19.9 ± 0.2	10.6 ± 0.8
	Token Deletion (Validity)	43 ± 3	16.1 ± 0.4	21.9 ± 0.6	12 ± 2
	Token Deletion (Protected)	49 ± 1	26 ± 2	20.4 ± 0.8	11.8 ± 0.3
	Atom Masking (Random)	40 ± 2	20 ± 2	23.7 ± 0.9	12.3 ± 0.3
	Atom Masking (Funct. Group)	39.1 ± 0.7	28 ± 2	27 ± 1	14.0 ± 0.4
	Bioisosteric Substitution	38 ± 2	16.0 ± 0.7	25.9 ± 0.5	22 ± 2
	Self-Training	33 ± 1	32.0 ± 0.5	32 ± 1	21 ± 2
	No Augmentation	46.3 ± 0.7	41 ± 2	24 ± 2	16 ± 1
MW	Enumeration	60.4 ± 0.3	38 ± 2	17 ± 2	14 ± 3
	Token Deletion (Random)	63.0 ± 0.7	49 ± 1	28.8 ± 0.6	12 ± 3
	Token Deletion (Validity)	57 ± 2	25 ± 1	29.7 ± 0.7	10 ± 1
	Token Deletion (Protected)	59 ± 1	31.6 ± 1.0	29 ± 1	8.9 ± 0.2
	Atom Masking (Random)	56 ± 1	31 ± 1	31.8 ± 0.7	9.4 ± 0.8
	Atom Masking (Funct. Group)	54 ± 1	39 ± 2	35.1 ± 0.7	12 ± 1
	Bioisosteric Substitution	57 ± 1	28.7 ± 0.5	31.3 ± 0.3	19 ± 2
	Self-Training	57.5 ± 0.4	51.1 ± 0.7	31.4 ± 0.4	17 ± 1
	No Augmentation	66.5 ± 0.9	56 ± 2	29.4 ± 0.9	12.3 ± 0.5
logP	Enumeration	45.0 ± 0.8	26 ± 2	28 ± 1	12 ± 2
	Token Deletion (Random)	44 ± 2	33 ± 2	22 ± 2	12 ± 4
	Token Deletion (Validity)	43 ± 2	19 ± 1	21.6 ± 0.4	10 ± 3
	Token Deletion (Protected)	41.7 ± 0.9	26.7 ± 0.7	25 ± 1	10 ± 2
	Atom Masking (Random)	44 ± 3	22 ± 3	25 ± 1	6.9 ± 0.5
	Atom Masking (Funct. Group)	43 ± 1	27 ± 3	29 ± 2	8 ± 1
	Bioisosteric Substitution	40 ± 2	22.6 ± 0.1	23.6 ± 0.8	9 ± 1
	Self-Training	36 ± 2	29.5 ± 0.2	22.3 ± 0.7	11.9 ± 0.5

	No Augmentation	40 ± 1	35.1 ± 1.0	23 ± 1	9 ± 2
**TPSA**	Enumeration	38 ± 2	38 ± 1	26.31 ± 0.01	23.7 ± 1.0
	Token Deletion (Random)	52.8 ± 0.9	47 ± 3	23.7 ± 0.5	23 ± 3
	Token Deletion (Validity)	47 ± 1	26.8 ± 0.5	24.7 ± 0.2	23 ± 1
	Token Deletion (Protected)	51 ± 1	35 ± 3	20.3 ± 0.5	23 ± 2
	Atom Masking (Random)	48 ± 3	29.4 ± 0.4	22.9 ± 0.9	19 ± 2
	Atom Masking (Funct. Group)	44 ± 3	36 ± 3	21 ± 1	16.5 ± 0.6
	Bioisosteric Substitution	48.7 ± 0.8	33 ± 2	23 ± 2	11 ± 1
	Self-Training	52 ± 1	52.5 ± 0.8	24.8 ± 0.9	19 ± 2
	No Augmentation	55.9 ± 1.0	53 ± 1	26 ± 2	17 ± 2

Supporting Table S8. We report the scaffold novelty of each target (PPARδ, PIM1, JAK2) of the training set with the compared to the starting fine-tune set at different sizes (10, 100) and similarity level (high, low) for following methods: Deletion (random, validity and protected), bioisosteric substitution and self-training. Enumeration and both random and functional group masking were not calculated, as they do not change the structure of the starting molecules.

Similarity	Target	Method	Scaffold Novelty for 10 fine-tuning molecules	Scaffold Novelty for 100 fine-tuning molecules
**High**	**PPARδ**	Token Deletion (Random)	7.5	3.5
		Token Deletion (Validity)	37.5	30.6
		Token Deletion (Protected)	12.5	8.4
		Bioisosteric Substitution	33.8	16.2
		Self-Training	78.8	45.6
	**PIM1**	Token Deletion (Random)	6.25	5.4
		Token Deletion (Validity)	42.5	50.1
		Token Deletion (Protected)	11.25	12.3
		Bioisosteric Substitution	15.0	17.8
		Self-Training	81.3	68.8
	**JAK2**	Token Deletion (Random)	10.0	3.0
		Token Deletion (Validity)	70.0	48.6
		Token Deletion (Protected)	16.3	12.9
		Bioisosteric Substitution	41.5	28.5
		Self-Training	76.3	70.6
**Low**	**PPARδ**	Token Deletion (Random)	5.0	6.0
		Token Deletion (Validity)	46.3	47.5
		Token Deletion (Protected)	15.0	12.9
		Bioisosteric Substitution	47.4	42.8
		Self-Training	82.5	59
	**PIM1**	Token Deletion (Random)	5.0	6.4
		Token Deletion (Validity)	57.5	60.6
		Token Deletion (Protected)	6.25	14.5
		Bioisosteric Substitution	35.1	34.5
		Self-Training	78.8	76
	**JAK2**	Token Deletion (Random)	6.3	5.1
		Token Deletion (Validity)	50.0	57.8
		Token Deletion (Protected)	15.0	16.0
		Bioisosteric Substitution	27.7	38.1
		Self-Training	82.5	74.1

References

1. Ertl, P., Altmann, E. & McKenna, J. M. The Most Common Functional Groups in Bioactive Molecules and How Their Popularity Has Evolved over Time. *J. Med. Chem.* **63**, 8408–8418 (2020).
